# Functional germline variants together with somatic mutations alter the integrity of cancer hallmark regulatory networks

**DOI:** 10.1186/s13073-026-01644-8

**Published:** 2026-04-29

**Authors:** Jiawei Dai, Mate Posta, Michal Marczyk, Tao Qing, Balazs Gyorffy, Lajos Pusztai

**Affiliations:** 1 Yale Cancer Center, Yale School of Medicine, 300 George Street, Suite 120, Rm 133, New Haven, CT 06511 USA; 2 Department of Bioinformatics, Semmelweis University, 1094 Budapest, Hungary; 3 Institute of Molecular Life Sciences, HUN-REN Research Centre for Natural Sciences, Budapest, Hungary; 4 Department of Data Science and Engineering, Silesian University of Technology, Gliwice, Poland; 5 Institute of Transdisciplinary Discoveries, University of Pecs, Pecs, Hungary

**Keywords:** Germline variant, Somatic mutation, Pathway disturbance, Malignant transformation, Phenotype convergence, Cancer heterogeneity

## Abstract

**Background:**

How germline and somatic alterations together contribute to alterations in cancer relevant pathways in individual cancers remains poorly understood. In this study, we provide a quantitative framework to characterize their contributions to pathway-level disturbances in individual cancers.

**Methods:**

We mapped germline and somatic DNA alterations of individual cancer samples from The Cancer Genome Atlas (TCGA), the Breast Cancer Genome Guided Therapy Study (BEAUTY) and the East Asian-ancestry Lung Adenocarcinoma (EAS-LUAD) cohort onto Gene Ontology (GO) and Kyoto Encyclopedia of Genes and Genomes (KEGG) pathways. To quantify pathway-level disturbances from germline and somatic origins, we calculated gene-level impact scores by integrating the functional importance of a gene in sustaining cell survival with the predicted impact of its variants. These scores were then used to compute a pathway disturbance score (CanSys score), that quantified the combined effect of gene-level alterations across all pathway members.

**Results:**

We found that more than 10% of TCGA cancers have rare protein function-altering germline alterations in cell cycle, telomere maintenance, and DNA repair hallmark pathways. At somatic mutation level, 12 pathways were altered in > 75% of cancers corresponding to core cancer hallmarks, however different genes within the same pathway are affected in different individuals, illustrating phenotypic convergence. In addition, 404 pathways were affected in ≥ 25% but < 50% of cancers which may contribute to the unique clinical course of each cancer. A freely available web tool (https://cansysplot.com) developed for this study enables visualization of GO/KEGG pathway disturbances from germline and somatic origins at the individual sample level.

**Conclusions:**

Our findings indicate that a substantial proportion of individuals with cancer harbor protein function-altering germline alterations in genes involved with cell cycle regulation, telomere maintenance, and DNA repair, whereas somatic mutations primarily affect pathways involved in cell adhesion, cell motility, metabolism, and a broad range of signal transduction processes. The unique combination of pathway alterations might explain the unique behavior of each cancer.

**Supplementary Information:**

The online version contains supplementary material available at 10.1186/s13073-026-01644-8.

## Background

Each cancer harbors hundreds of somatic DNA alterations that have accumulated against the background of thousands of germline variants [1, 2]. Many germline variants in coding regions are predicted to alter protein function or influence gene expression levels, and these germline differences between individuals contribute to variations in individual features and susceptibilities to diseases illustrating the functional importance of the combined effect of non-pathogenic variants. During aging, somatic mutations and epigenetic changes accumulate against these inherited variations in gene functions. We previously showed that individuals who develop cancer at a younger age have greater number of high functional impact germline variants in hundreds of cancer biology-related genes and fewer somatic mutations in these genes than those who develop cancer later in life [3]. These germline variants in cancer-related genes included both common and rare polymorphisms, none of which were linked to cancer risk in genome-wide association studies, suggesting context dependent effect. A recent study also suggested that a very large number of genes may contribute to cancer biology through their first- and second-degree interactions with canonical cancer genes [4]. It is also increasingly recognized that individually rare and non-recurrent, “long-tail” somatic mutations that were previously considered biologically irrelevant passenger mutations have biological impact in cancer [5]. We hypothesize that it is the combined impact of the many functional germline variants, with individually subtle effects, and acquired recurrent and non-recurrent somatic mutations, also with variable functional importance, collectively destabilize the integrity of cancer hallmark pathways in ways that are unique to each individual leading to malignant transformation but also to unique clinical course of each cancer [6].

Our goal was to uncover disruptions in key biological processes involved in cancer biology, and to reveal sample-specific biological pathway disturbances by quantifying the contributions of germline and somatic alterations to pathway-level disturbances at the single-sample level. To measure the extent of pathway disturbance, we first calculated gene-level impact scores by integrating the gene’s functional importance in sustaining cell survival with the predicted impact of all variants it harbors, and subsequently computed a pathway disturbance score, CanSys, that quantifies the combined effect of gene-level alterations across all pathway members. Three cancer datasets from The Cancer Genome Atlas (TCGA), the Breast Cancer Genome Guided Therapy Study (BEAUTY) and the East Asian-ancestry Lung Adenocarcinoma (EAS-LUAD) cohort were used in this study. We also built a visualization tool that displays individual sample-level results in the framework of Gene Ontology (GO) or the Kyoto Encyclopedia of Genes and Genomes (KEGG) pathways with a public facing interface (https://cansysplot.com) that allows investigators to apply CanSys to their own data.

## Methods

### Calculation of the pathway alteration scores

The input data includes germline and somatic Variant Call Format (VCF) files and an optional mRNA expression matrix in text format (txt) from individual samples. The expression data, if provided, is used to filter genes that are not expressed in the sample. We assign expressed versus not-expressed status to each gene using the dpGMM R package (version 0.1.8) that fits a Gaussian mixture model to determine a cutoff value for the expression status [7]. To optimize the GMM model fitting, the mRNA expression data is log2(count + 1) transformed for input, and the estimated number of model components (KS) is set to 5. The cutoff values are derived using the k-means algorithm (k = 2), where an argument of the clustering procedure is a matrix (size KS by 3) containing all parameters of the Gaussian mixture model. As a result, Gaussian components are divided into two groups, and the cutoff expression value between them is estimated with the maximum a posteriori probability rule. Genes with expression lower than the smallest cutoff are considered not expressed, since they mostly represent technical artifacts of sampling or sequencing errors rather than true biological expression.

To calculate gene-level impact scores, we first annotate the single nucleotide variants (SNVs) and insertion/deletions (INDELs) for predicted functional importance using the Phred-scaled scores from the Combined Annotation Dependent Depletion (CADD, https://cadd.gs.washington.edu/, version 1.7) [8, 9]. CADD includes scores for 8,812,917,337 SNVs and 105,496,260 INDELs. CADD scores can range from 0 to 99, the higher the value the greater the predicted impact on gene function. Our method allows the selection of the desired CADD score cutoffs for deleteriousness. The gene-level CADD score for gene $g$ harboring $N$ SNVs and INDELs (that each meet the selected CADD threshold) is calculated as the average of the CADD scores and scaled as shown below:

$$gene{CADD score}_{g}=\frac{{\sum }_{n=1}^{N}{CADD score}_{n}}{100N}$$

Pre-calculated deleteriousness metrics were also obtained from AlphaMissense and VARITY for comparison with CADD [10, 11]. Next, we assign functional importance to a gene by calculating the average cancer type-specific CRISPR dependency scores from the Cancer Dependency Map (DepMap, https://depmap.org/portal/, version Public 23Q2) using data from the relevant cancer cell lines [12]. The dependency scores measure the impact of gene knockout on cell viability in vitro across hundreds of different cancer cell lines and are expressed as a scaled value between 0 (no impact on cell survival) to 1 (survival essential). For common cancer types, we precalculated cancer-type specific DepMap scores for each gene (Additional file 1: Table S1). We calculate the gene-level impact score for gene g by multiplying the variant-importance and gene-importance scores:

$${geneImpact score}_g={geneCADD score}_g\cdot{geneDepMap score}_g$$

The higher the gene-level impact score, the greater the predicted functional impact of the genomic alteration. If expression data is provided, the impact scores for unexpressed genes are set to 0.

We map gene-level impact scores to biological pathways and calculate a pathway genomic alteration enrichment score (ES) by applying a modified Gene Set Enrichment Analysis (GSEA) method. We replace gene expression fold changes, traditionally used in GSEA, with gene-level impact scores to calculate ES. Genes are sorted from highest to lowest values of gene-level impact scores, and then a running sum for ES is calculated for each gene (Hit: gene is in pathway S; Miss: gene is not in S). The maximum value of the running sum for ES is taken as the ES for pathway S, denoted as $ES\left(S\right)$:

$${P}_{Hit}\left(S,i\right)=\sum_{{gene}_{j}\in S,j\le i}\frac{{impact score}_{j}}{{impact score}_{total}}$$

$${P}_{Miss}\left(S,i\right)={\sum }_{{gene}_{j\notin S,j\le i}}\frac{1}{N-{N}_{S}}$$

$$ES\left(S\right)=ma{x}_{i=1}^{N}\left({running sum for ES}_{i}\right)=ma{x}_{i=1}^{N}\left(\left|{P}_{Hit}\left(S,i\right)-{P}_{Miss}\left(S,i\right)\right|\right)$$

where $i$ is the rank of the gene, $impact {score}_{j}$ is the gene-level impact score for the gene ranked at $j$ (where $j\le i$), ${impact score}_{total}$ is the sum of gene-level impact scores for all genes, $N$ is the total number of expressed genes, and ${N}_{S}$ is the number of genes in pathway S. To rapidly assess the significance of gene alteration enrichment for pathway S, the fgseaMultilevel() function from the fGSEA R package (version 1.24.0) is utilized [13]. This function enables the fast generation of null distributions for ES by randomly permuting genes to create random gene sets that match the same size of the pathway. The scoreType parameter is set as “pos”. The number of permutations for pathways affected by somatic mutations is set at 6,000, achieving median average pairwise Pearson correlations of *P*-values per TCGA breast sample across 10 runs of 0.9967 for GO and 0.9935 for KEGG. For pathways affected by germline mutations, the number is set at 1,000, resulting in median average pairwise Pearson correlations per sample across 10 runs of 0.9910 for GO and 0.9843 for KEGG. The output includes $ES\left(S\right)$ normalized to the mean of the permutation-derived ESs, referred to as the normalized ES for pathway S $NES\left(S\right)$), along with the Benjamini-Hochberg (BH) adjusted *P*-value. Pathways with adjusted *P* < 0.25 are considered genomically disturbed/altered.

Since the normalized ESs were designed to compare pathways within a single sample, to quantify the affected pathways that can also be used for comparisons between samples, we compute a Cancer Systems integrity score/pathway disturbance score (CanSys score) for each significantly enriched pathway S by multiplying the $NES\left(S\right)$ and the average gene-level impact scores within pathway S:

$$CanSys score(S)=NES(S)\cdot \frac{{\sum }_{{gene}_{j}\in S}{impact score}_{j}}{{N}_{S}}$$

### Human biological pathways relevant to cancer biology

Human biological pathways were taken from the "Biological Process" category of GO (https://geneontology.org/), and KEGG (https://www.kegg.jp/kegg/) [14–16]. GO gene annotations (GO terms) are based on gene functional relationships. Gene membership overlap occurs through the child-parent relationships in hierarchically nested GO terms where child node terms are more specific than their parent terms. We used the Pronto Python library (https://zenodo.org/records/8255798, version 2.5.5) to parse the fully axiomatized version of the GO, enabling the extraction of terms along with their corresponding classifications "Biological Process", "Molecular Function" and "Cellular Component". The gene lists corresponding to 15,283 GO terms related to "Biological Process" were retrieved from the AmiGO2 website (https://amigo.geneontology.org/). A mapping of cancer hallmarks to GO processes was previously presented [17].

The KEGG gene annotation assigns genes that participate in the same biological process into biological pathways. However, the same gene can be assigned to multiple different pathways. From the 355 human pathways in KEGG, we selected 176 pathways involved in "Metabolism", "Genetic Information Processing", "Environmental Information Processing" and "Cellular Process" based on their known cancer relevance. KEGG pathways categorized under "Organismal Systems", "Human Diseases", and "Drug Development" were excluded. This allows us to focus on the fundamental cellular and molecular mechanisms underlying oncogenesis while avoiding redundancy from disease- or drug-oriented annotations and ensuring mechanistic interpretability. Gene lists for these KEGG pathways were extracted from the KEGG API (https://www.kegg.jp/kegg/rest/keggapi.html).

### Visualization of pathway anomalies

Two network spaces were constructed from selected GO and KEGG pathways, respectively, to visualize sample-level pathway anomalies in the standard relational framework of these databases. Within these networks, each node represents a distinct biological pathway, and the node size is determined by the natural logarithm of the number of genes it contains. Nodes are connected by edges if there is direct relationship between them in the databases. For the GO network, 34,080 directional connections among 15,283 GO terms were extracted using the Pronto Python library, including five types of relationships: "is a", "part of", "regulates", "positively regulates", and "negatively regulates". For the KEGG, the KEGG API was used to obtain the relational information for connected pathways. A total of 714 non-directional connections were found among the 176 KEGG pathways. To enhance the representation of significant nodes, our web tool visualizes GO networks by prominently displaying significant nodes. Non-significant nodes are included only if they lie on the path between a significant node and the root node (GO:0008150, Biological Process).

We developed a web-based application using Flask (version 3.0.3) to enable custom analysis and visualization of VCF files and pathway networks. The web application was developed using the Flask microframework (version 3.0.3) for Python (version 3.11.1). Flask was chosen for its flexibility, lightweight nature, and suitability for building web applications with dynamic content. The application follows a Model-View-Controller (MVC) architectural pattern. The front-end interface was built using standard web technologies: HTML5 for structuring content, CSS3 for styling, and JavaScript for client-side interactivity. Cytoscape.js (version 3.29.2) was integrated as the primary library for interactive network visualization within the web browser.

Two types of files can be uploaded through the web interface: VCF files and expression files. The VCF files contain information about genomic variations. The application expects VCF files conforming to the standard VCF format aligned to the reference genome (GRCh38). The expression files contain gene expression data, which can be used to integrate gene expression information into the pathway analysis. The expected format for the expression files is a tab-separated file with gene identifiers in the first column and expression values in subsequent columns.

Uploaded files are stored temporarily in a dedicated server. A filtering criterion based on an adjusted *P*-value (default: BH adjusted *P* < 0.25) was applied to identify significant GO and KEGG terms. To explore the GO or KEGG networks, a breadth-first search (BFS) algorithm was implemented, starting from these significant terms and traversing relevant nodes and their successive layers of related terms to generate a subgraph. The generated subgraphs were converted into a format suitable for Cytoscape.js and rendered within the web browser. Each node in the subgraph represents a GO term or KEGG pathway. Node size was dynamically adjusted based on the CanSys scores derived from the analysis results, with larger nodes indicating a higher disturbance level. Nodes are color-coded based on statistical significance with red nodes marking significant terms and grey nodes marking non-significant terms. Edges represent relationships between GO terms or KEGG pathways. Edge colors were assigned according to the relationship type (e.g., "is-a", "part-of", "negatively regulates", "positively regulates", and "regulates" for GO; "Related" for KEGG). Cytoscape.js enables interactive exploration of the network, including zooming, panning, node selection, and displaying node information on hover. Various layout algorithms (standard and Euler) are available to optimize the graph layout for improved readability. The Python package networkx (version 3.1) was used to represent and manipulate the GO and KEGG pathway networks as graph objects.

The application provides downloadable output files containing the results of the pathway enrichment analysis for both GO and KEGG pathways. These files are generated in tab-delimited text format, facilitating easy import into spreadsheet software or other data analysis tools.

Uploaded files and analysis results are stored temporarily on the server's file system within session-specific locations, allowing analyses can be revisited using the unique session ID. Precalculated results from TCGA data are stored in a server’s directory structure for demonstration purposes.

### Clinical datasets

We applied CanSys to three cancer datasets including TCGA [18], BEAUTY [19] and EAS-LUAD [20]. From the TCGA, somatic VCFs of whole exome sequencing (WES) and RNA-seq of the primary cancer samples were downloaded and germline variants were obtained from a previous publication [2]. We collected a total of 9,384 somatic VCFs and 9,297 germline VCFs across 31 cancer types with matching gene expression data (Additional file 1: Table S2). The estrogen receptor (ER) status and the human epidermal growth factor receptor 2 (HER2) of breast cancer samples was assigned based on immunohistochemistry (IHC) results using the ASCO-CAP definitions. When IHC results were not available, ER status was assigned based on *ESR1* mRNA expression level as previously described [21]. HER2 IHC score of 2 + was considered equivocal, defined as weak to moderate complete membrane staining observed in > 10% of invasive tumor cells. This means the protein expression is neither clearly negative (IHC 0/1 +) nor strongly positive (IHC3 +).

The BEAUTY cohort included tumor and germline WES and tumor whole transcriptome RNA-seq data from 118 Stage I-III breast cancers. We found that 7 WES files were damaged and therefore we only included 111 cancers in our analysis. Sequence data in FASTQ format were acquired using the prefetch and the fastq-dump tools of the Sequence Read Archive (SRA) Toolkit (https://github.com/ncbi/sra-tools, version 3.0.2). The EAS-LUAD cohort included similar matched sequencing data from 169 lung adenocarcinoma (LUAD) patients of East Asian-ancestry. Sequence data in FASTQ format were acquired using pyEGA3 (https://github.com/EGA-archive/ega-download-client, version 5.2.0). Quality control and adapter trimming were executed using Trim Galore (version 0.6.10) with default parameters [22]. Subsequently, for the WES reads, alignment of the cleaned reads to the human reference genome (GRCh38) was conducted with the Burrows–Wheeler Aligner (BWA, version 0.7.17-r1188) [23]. Duplicate reads were marked and removed via the Picard tool of the Genome Analysis Toolkit (GATK, version 4.4.0.0) [24]. Base quality score recalibration was achieved using GATK's BaseRecalibrator and ApplyBQSR tools. Somatic variants were detected using GATK's Mutect2 tool for tumor samples with matched normal samples, with subsequent variant filtration through GATK's FilterMutectCalls tool. Germline variant detection in normal samples was performed using VarScan2 (version 2.4.6) [25]. For the transcript reads, mRNA levels were quantified using the Spliced Transcripts Alignment to a Reference (STAR, version 2.7.11a) in two-pass mode [26].

The somatic variants of the cancer samples were filtered for allelic depth (AD) ≥ 5 and variant allele frequency (VAF) > 5%, and germline variants were filtered with AD ≥ 5 and VAF > 20%. ANNOVAR (version Date: 2020–06–08) was utilized to annotate population allele frequencies from the Genome Aggregation Database (gnomAD, version 4.1) and clinical pathogenicity from ClinVar (version Date: 2025–07–21) [27–29]. These annotations were used to evaluate the effectiveness of using CADD scores in quantifying variant deleteriousness and filtering out common variants with potentially modest functional impact. Finally, for germline variants, we retained only those with a gnomAD population allele frequency ≤ 0.001 and a CADD score ≥ 15, and considered these variants deleterious when calculating gene-level impact scores for the CanSys score calculation. This CADD threshold represents the median value for all possible canonical splice site changes and non-synonymous variants in CADD v1.0.

### Population reference for background variation

As a population-based reference for germline pathway alterations in individuals not diagnosed with cancer at the time when they donated normal tissue, we used data from 2,504 unrelated individuals included in the 1000 Genomes Project (1kGP; https://www.internationalgenome.org/data-portal/data-collection/30x-grch38) [30]. We restricted the analysis to exonic variants and excluded variants with a gnomAD population allele frequency > 0.001 or a CADD score < 15. For each individual, only non-reference genotypes (0/1 or 1/1) were considered as carrying a variant. These variants were then mapped to genes and pathways and scored using the same cancer-type–specific DepMap weights and TCGA expression-based filters as in the TCGA cohorts, ensuring a symmetric comparison between cancer patients and non-cancer individuals. The number of permutations was set to 1,000. For cancer types that occur only, or almost exclusively, in a single sex (e.g., breast, cervical, ovarian, endometrial, and uterine cancers in females; prostate and testicular cancers in males), results were evaluated using 1kGP individuals of the corresponding sex.

To assess pathway-level differences between TCGA and 1kGP while accounting for cohort effects, we performed a residual-based analysis. The percentage of samples with significantly affected pathways in TCGA was modeled as a function of that in 1kGP using linear regression, and residuals were calculated as the difference between observed and predicted values. Pathways were then ranked by residuals to highlight those with the most distinct alteration patterns between TCGA and 1kGP.

### Quantification of phenotypic convergence

For a given pathway S, let ${k}_{g}$ denote the number of individuals in which gene $g\in S$ is altered. The convergence score was defined as $C\left(S\right)$:

$$C\left(S\right)={\sum }_{g\in S}\frac{{k}_{g}\left({k}_{g}-1\right)}{2}$$

Which corresponds to the total number of sample pairs that share alterations in genes within the pathway. The score was then normalized by the number of genes in the pathway and by the total number of possible sample pairs. To evaluate significance, we generated 1,000 randomized gene sets of the same size as S by sampling from the genome-wide gene universe. Convergence scores from these randomized sets formed an empirical null distribution, from which an empirical *P*-value and a Z-score were obtained. The *P*-value assesses whether the observed convergence exceeds random expectation, whereas the Z-score quantifies the magnitude of this non-random phenotypic convergence.

### Comparison with the DAVID functional annotation

For CanSys results, Fisher’s exact test was used to identify differentially affected GO and KEGG pathways between ER-positive (*n* = 808) and ER-negative (*n* = 246) TCGA breast cancer samples, as well as between LUAD (*n* = 526) and lung squamous cell carcinoma (LUSC; *n* = 494) TCGA samples at the somatic mutation level. Pathway anomalies were first determined using a BH adjusted *P* < 0.25 in permutation testing. Fisher’s exact test *P*-values were subsequently adjusted using the BH method, and pathways with adjusted *P* < 0.05 were then considered significantly differentially affected.

Differential mutation analysis was performed for the same group comparisons using Fisher’s exact test to identify genes with significantly different somatic mutation frequencies. Genes with BH adjusted *P* < 0.05 were used as input for functional annotation using the Database for Annotation, Visualization, and Integrated Discovery (DAVID; https://davidbioinformatics.nih.gov/home.jsp) [31]. The EASE Threshold and count threshold were set to the default 0.1 and 2, respectively. Functional categories, including GO terms related to "Biological Process" and KEGG pathways involved in "Metabolism", "Genetic Information Processing", "Environmental Information Processing" and "Cellular Process", were retained. *P-*values were adjusted using the BH method, and pathways with adjusted *P* < 0.05 were considered significantly disturbed. The resulting ER-associated pathways were compared with those identified by CanSys.

### Statistical analysis

QQ plots were generated to assess whether the pathway-level *P*-values produced by CanSys were well calibrated under the uniform null expectation. TCGA samples with different numbers of significantly affected pathways were randomly selected, and their observed –log_10_ (*P*) values were compared with the expected values under a uniform null distribution. Hierarchical clustering of CanSys scores in breast cancer samples was performed using the hclust function in R, employing the "Euclidean" distance metric and setting k to 3 to ensure a suitable sample size in each cluster. Fisher’s exact test was used to identify pathways that were significantly differentially affected between clusters of samples. To control the false discovery rate, the BH method was used. Pathways with a BH adjusted *P* < 0.05 were considered significantly differentially affected. Statistical analyses also included the Wilcoxon rank-sum test to compare deleteriousness scores, age at diagnosis, and Fisher’s exact test to compare cancer stage, ER status, and HER2 status between clusters of samples. Across all tests, a two-sided *P* < 0.05 was considered statistically significant. The log-rank test was used for statistical analysis of survival curves estimated using Kaplan–Meier method. All analyses and visualizations were performed using R (version 4.3.1).

## Results

### The CanSys architecture

**Fig. 1:**
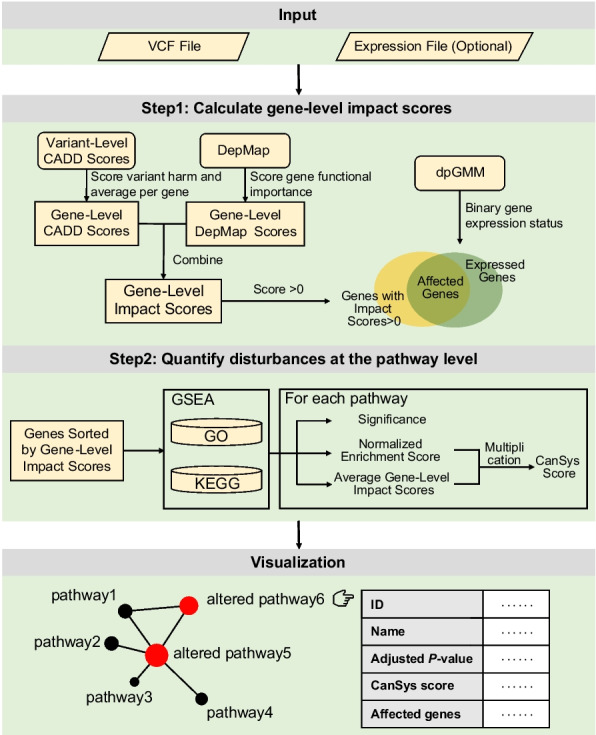
Overview of CanSys. CanSys requires a VCF file aligned to the reference genome (GRCh38) as input, with the option to include an mRNA expression file. The tool calculates gene-level impact scores, by integrating cumulative variant-level CADD scores that capture the impact of gene alterations on gene function and a gene-importance measure from DepMap that reflects the importance of the gene in cell survival. Optional, gene expression data input refines these scores, by allowing to set the impact scores for non-expressed genes to 0. Affected genes are mapped to GO and KEGG pathways, and a modified gene set enrichment analysis identifies significantly altered pathways. The final CanSys score for a pathway is a composite of the normalized enrichment score and the average gene-level impact scores within the pathway. The sample-level results for all significantly affected pathways are visualized in the standard relational framework of the GO and KEGG pathway databases

Figure 1 illustrates the overall CanSys calculation and visualization process. Starting from a VCF file aligned to the reference genome (GRCh38), CanSys integrates variant- and gene-level information to quantify functional disruptions in individual samples. Each gene receives an impact score derived from both the cumulative CADD scores of its variants, reflecting the impact of gene alterations on gene function, and a gene essentiality score from DepMap, representing its importance in cell survival. When available, expression data are incorporated to suppress the contribution of genes that are transcriptionally inactive in the sample. These gene-level impact scores are mapped onto GO and KEGG pathways by performing a modified GSEA, which identifies biological processes significantly affected by genetic alterations. Finally, a pathway disturbance score, termed the CanSys score, is calculated as a composite of the normalized enrichment score and the average gene-level impact scores within the pathway is calculated. The sample-level results for all significantly affected pathways are visualized in the standard relational framework of the GO and KEGG pathway databases.

We used pre-calculated CADD scores to quantify variant deleteriousness because of their genome-wide coverage across all variant types. An analysis of all possible nonsynonymous SNVs in ten frequently mutated DNA repair genes (*ATM*, *BRCA2*, *PRKDC*, *ATR*, *BRCA1*, *MSH6*, *POLE*, *FANCM*, *FANCD2*, and *SLX4*; *n* = 170,427) demonstrated that, while CADD shows comparable discriminative power to other deleteriousness metrics such as AlphaMissense and VARITY in distinguishing ClinVar-annotated benign and pathogenic variants, it provides substantially broader variant coverage (Additional file 2: Fig. S1). Further analysis of all annotatable variants in the exonic regions of two frequently amplified cancer-associated chromosomal bands 10q23 and 17p13 (*n* = 35,336 and 106,355, respectively) confirmed that applying a CADD cutoff in combination with population allele frequencies effectively filters out benign or likely benign common variants (Additional file 2: Fig. S2).

The distribution of CADD scores and DepMap scores for genes expressed in breast cancer (BRCA) in the TCGA are shown on Additional file 2: Fig. S3. The most frequently observed variant-level CADD score was 0.243 for somatic variants, and 0.00675 for germline variants (Additional file 2: Fig. S3A). The most frequent gene-level CADD scores were lower when somatic variants were used to calculate these scores (0.000144) than when germline variants were used (0.00153) due to the cumulative effect of the much larger number of germline variants (Additional file 2: Fig. S3B). Gene-level impact scores that combine functional importance of the gene with gene-level CADD score are low when the gene-level CADD scores are low even if the gene DepMap score is high, to reflect that while the gene is functionally important, its biological function is not substantially altered by the detected variant (Additional file 2: Fig. S3C and D). We also assessed the impact of selecting different CADD score thresholds to define variant-level importance, and different *P*-value thresholds to define significantly altered pathways. While applying a CADD filter (score ≥ 10) markedly altered the metrics compared to the unfiltered baseline (CADD cutoff = 0), the results—including average gene-level impact scores and the number of significantly altered pathways—remained relatively stable across the 10 to 20 threshold range (Additional file 2: Fig. S4). Consequently, after excluding germline variants with a gnomAD population allele frequency > 0.001, we applied a CADD threshold of ≥ 15 to calculate gene-level impact scores for the CanSys score computation. The QQ plots in Additional file 2: Fig. S5 show the inflation pattern of the pathway-level test statistics obtained from CanSys for three randomly selected TCGA samples with varying numbers of significantly affected pathways. Most pathways fall closely along the diagonal, indicating well-calibrated null distributions. Only a small subset of highly significant pathways shows upward deviation corresponding to the varying numbers of significantly affected pathways across the samples.

### Interactive network visualization tool

**Fig. 2:**
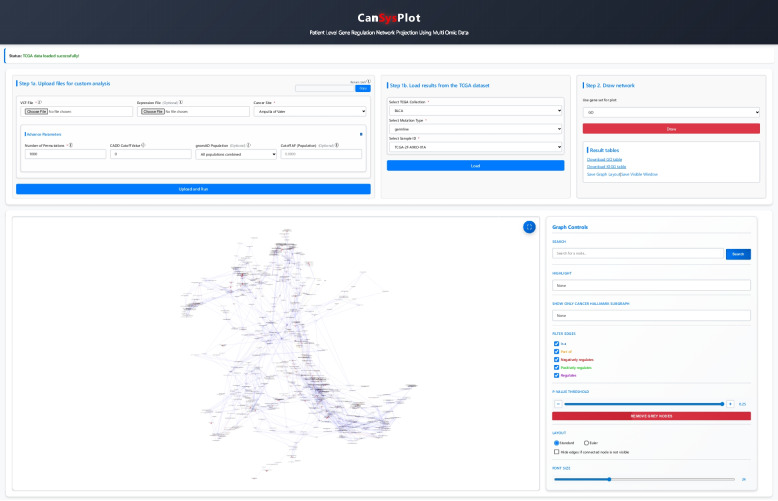
Interactive network visualization tool. The web-based application enables the analysis and visualization of pathway disturbances in GO or KEGG networks. VCF files are uploaded and processed through a streamlined analysis pipeline integrated into the platform. The application also allows for the filtering of significant GO and KEGG terms based on a custom adjusted *P*-value cutoff, facilitating an in-depth exploration of related biological pathways. Utilizing Cytoscape.js for visualization, the platform allows users to interact with the generated subgraphs, with nodes color-coded by their statistical significance. Additionally, the application supports the visualization of disturbance networks using data derived from TCGA

The web-based application, https://cansysplot.com, enables the analysis and visualization of custom VCF files and map sample-level alterations onto GO or KEGG networks (Fig. 2). VCF and expression files can be uploaded and processed through a streamlined analysis pipeline that allows for either default or customized parameters, including the number of permutations, CADD cutoff value, and gnomAD population allele frequency threshold. Within the networks, specific nodes can be searched, daughter nodes of a main GO category can be highlighted, and cancer hallmark pathways can be selectively displayed. The application also supports filtering of significant GO and KEGG pathways based on a customizable adjusted *P*-value cutoff, facilitating in-depth exploration of related biological pathways. Visualization is implemented using Cytoscape.js, providing interactive subgraphs with nodes color-coded according to their statistical significance. Embedded links are available to analyze data from TCGA directly.

### Pan-cancer pathway alterations identified by CanSys in the TCGA

Table 1 shows the number of significantly affected GO pathways across all cancers in TCGA as function of *P*-value thresholds. When only somatic alterations were used to identify altered pathways with BH adjusted *P* < 0.25, we found 12 pathways (cellular process, cellular component organization or biogenesis, cellular component organization, developmental process, biological regulation, anatomical structure development, regulation of biological process, regulation of cellular process, multicellular organismal process, positive regulation of biological process, multicellular organism development, and positive regulation of cellular process) that were altered in ≥ 75% of samples, 61 were altered in ≥ 50 but < 75%, 404 in ≥ 25% but < 50%, and 14,806 in at least one sample. When germline alterations were used to identify altered pathways (BH adjusted *P* < 0.25), 2 pathways (cellular process and cellular component organization or biogenesis) were altered in ≥ 75% of samples, 7 (cellular component organization, metaphase/anaphase transition or mitotic cell cycle, metaphase/anaphase transition of cell cycle, anaphase-promoting complex-dependent catabolic process, protein K11-linked ubiquitination, developmental process, and anatomical structure development) altered in ≥ 50 to < 75%, 60 in ≥ 25% to < 50%, and 15,214 in at least one sample. The frequently shared germline affected pathways across cancer types suggests that individuals who develop cancer already harbor subtle pathway alterations at birth. While the number of affected pathways changes depending on *P*-value thresholds, these results show that cancers of different histologic types broadly share alterations in a set of common pathways indicating that a core set of biological processes must be altered for malignant transformation, regardless of tissue of origin. In addition, each cancer also harbors a variable number of unique pathway alterations. These large and varied number of pathways that are altered only in a few samples might contribute to the unique biological behavior of each cancer.

**Table 1:** Number of significantly affected GO pathways across cancers in TCGA as function of nominal *P*-values and false discovery rate adjusted *P*-value thresholds

Affected Type	Significance threshold	Number of GO pathways			
		affected < 25% of the samples	affected ≥ 25% but < 50% of the samples	affected ≥ 50% but < 75% of the samples	affected ≥ 75% of the samples
Somatic- affected	*P* < 0.05	15,173	99	11	0
	*P* < 0.1	14,984	258	38	3
	*P* < 0.25	13,634	1502	105	42
	BH adjusted *P* < 0.05	15,283	0	0	0
	BH adjusted *P* < 0.1	15,268	15	0	0
	BH adjusted *P* < 0.25	14,806	404	61	12
Germline- affected	*P* < 0.05	15,046	194	31	12
	*P* < 0.1	14,736	405	111	31
	*P* < 0.25	12,903	1993	267	120
	BH adjusted *P* < 0.05	15,278	4	1	0
	BH adjusted *P* < 0.1	15,275	7	1	0
	BH adjusted *P* < 0.25	15,214	60	7	2
Overlapping both somatic- and germline- affected	*P* < 0.05	15,270	10	3	0
	*P* < 0.1	15,243	28	11	1
	*P* < 0.25	15,118	115	38	12
	BH adjusted *P* < 0.05	15,283	0	0	0
	BH adjusted *P* < 0.1	15,282	1	0	0
	BH adjusted *P* < 0.25	15,269	11	3	0

*BH* Benjamini–Hochberg procedure

Since GO and KEGG employ different rules to assign genes into biological processes, we also assessed pathway alterations using the KEGG annotation. The overall pattern of results was similar, but fewer pathways were identified as significantly altered (Table 2). This difference is due to the much larger GO ontology space (there are 15,283 GO terms encompassing 17,510 unique genes) compared to the 176 KEGG pathways (*n* = 6,236 unique genes). Pathway size shows a positive correlation with the percentage of samples that show significant alteration in a given pathway when somatic mutations are considered. Weaker correlation is seen in the germline analyses (Additional file 2: Fig. S6). This observation likely reflects the distinct distributions of the two variant landscapes: somatic mutations are few in number but highly dispersed across many genes, and therefore larger pathways cover more genomic space in which these mutations can occur. In contrast, germline variations are numerous but are limited to a much smaller subset of genes, making pathway size less relevant.

**Table 2:** Number of significantly affected KEGG pathways across cancers in TCGA as function of nominal *P*-values and false discovery rate adjusted *P*-value thresholds

Affected Type	Significance threshold	Number of KEGG pathways			
		affected < 25% of the samples	affected ≥ 25% but < 50% of the samples	affected ≥ 50% but < 75% of the samples	affected ≥ 75% of the samples
Somatic affected	*P* < 0.05	175	1	0	0
	*P* < 0.1	165	11	0	0
	*P* < 0.25	125	51	0	0
	BH adjusted *P* < 0.05	176	0	0	0
	BH adjusted *P* < 0.1	176	0	0	0
	BH adjusted *P* < 0.25	157	19	0	0
Germline affected	*P* < 0.05	165	8	3	0
	*P* < 0.1	159	12	5	0
	*P* < 0.25	111	56	7	2
	BH adjusted *P* < 0.05	176	0	0	0
	BH adjusted *P* < 0.1	176	0	0	0
	BH adjusted *P* < 0.25	171	5	0	0
Overlapping both somatic and germline affected	*P* < 0.05	176	0	0	0
	*P* < 0.1	176	0	0	0
	*P* < 0.25	174	2	0	0
	BH adjusted *P* < 0.05	176	0	0	0
	BH adjusted *P* < 0.1	176	0	0	0
	BH adjusted *P* < 0.25	176	0	0	0

*BH* Benjamini–Hochberg procedure

The top 50 most frequently significantly affected GO and KEGG pathways (BH adjusted *P* < 0.25) in the TCGA are shown on Additional file 2: Fig. S7 and Fig. S8. The GO pathways of cellular process, cellular component, development, regulation, metabolism and cell differentiation are almost uniformly affected by somatic mutations in all cancer types (Additional file 2: Fig. S7A). At the germline level, cellular process and cellular component pathways are affected uniformly across cancer types; meanwhile, glioblastoma multiforme (GBM), kidney renal clear cell carcinoma (KIRC), acute myeloid leukemia (LAML), and mesothelioma (MESO) showed distinct patterns among the top-ranked GO pathways (Additional file 2: Fig. S7B). Many pathways are affected by both germline and somatic alterations (Additional file 2: Fig. S7C). Similar analysis of KEGG pathways, showed that somatic alterations primarily affected genes involved in cytoskeleton and cell adhesion, and a broad range of signal transduction processes (Rap1, mTOR, FoxO, MAPK, ErbB, PIK3-Akt, p53), affecting 20% to 30% of samples in the pooled analysis of all cancers while specific cancer types showed lower alteration frequencies (Additional file 2: Fig. S8A). Pancreatic adenocarcinoma (PAAD), thyroid carcinoma (THCA) and uterine carcinosarcoma (UCS), had the highest percentage of samples with somatic mutation driven pathway alterations. Uveal melanoma (UVM) showed a unique pattern of pathway disturbances characterized by unusually high frequency of somatic alterations in calcium signaling, gap junction, and cGMP-PKG signaling pathways (91.2–96.2% of samples). At the germline level, patients with cancer in the TCGA carry alterations in genes involved in cell cycle regulation, RNA processing, DNA replication, and DNA repair (Additional file 2: Fig. S8B). In the non-cancer 1kGP cohort, we also observed variants affecting similar pathways in many individuals (Additional file 2: Fig. S9A and B). Given the technical differences between the two cohorts, we assessed the overall consistency of the alteration patterns between the two cohorts using correlation analysis. The correlations in the percentage of samples with pathway significantly affected between TCGA and 1kGP were moderate, at 0.55 and 0.67 for GO and KEGG pathways, respectively. For CanSys scores, the correlations were 0.45 and 0.68. Residual-based analysis identified pathway-specific differences between TCGA and 1kGP (Additional file 1: Tables S3 and S4). GO pathways enriched in TCGA were predominantly related to mitotic cell cycle progression, microtubule dynamics, and chromosome segregation, whereas those enriched in 1kGP were largely associated with RNA metabolism, transcriptional regulation, and ribosome biogenesis. Similarly, KEGG pathways with positive residuals in TCGA included cell cycle, DNA repair, ubiquitin mediated proteolysis, and transcriptional and translational processes. In contrast, pathways with negative residuals were largely associated with metabolic processes, immune signaling, and cellular homeostasis, including pathways such as apoptosis, necroptosis, and oxidative phosphorylation.

**Fig. 3:**
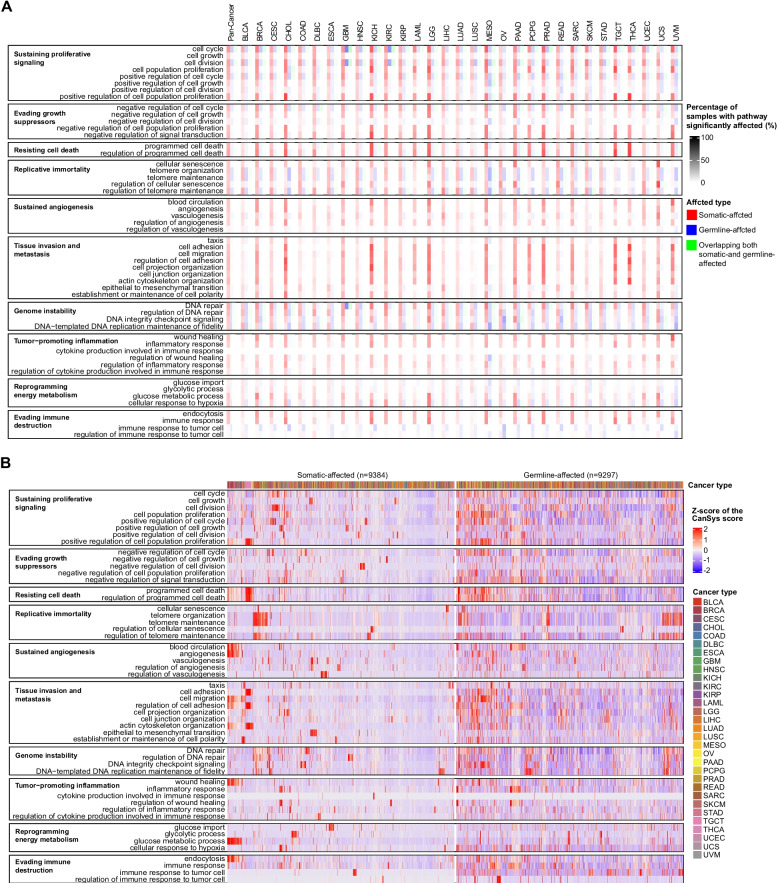
Alteration prevalence in 52 GO terms corresponding to 10 cancer hallmarks in TCGA cancer types. **A** The heatmap shows the percentage of samples that are significantly affected (BH adjusted *P* < 0.25) in various cancer hallmark pathways in each cancer type. Color coding indicates the origin of the alterations, red: somatic, blue: germline, and green: both. **B** The heatmap shows the CanSys scores for the cancer hallmark pathways at the somatic and germline level, respectively. Columns (samples) are ordered based on hierarchical clustering. Germline variants were excluded if they had a gnomAD population allele frequency > 0.001 or a CADD score < 15. The Z-scores of the CanSys scores were capped at a range from −2 to 2. BH: Benjamini–Hochberg procedure

To facilitate biological process level interpretation of pathway alterations, we also performed an analysis using only 52 GO terms that cover 10 cancer hallmarks [17]. The frequency of pathway alterations due to somatic mutations varied across cancer types (Fig. 3A); cholangiocarcinoma (CHOL), kidney chromophobe (KICH), brain lower grade glioma (LGG), MESO, prostate adenocarcinoma (PRAD), THCA, and testicular germ cell tumors (TGCT) had the most frequent and diverse somatic mutation driven pathway alterations in growth suppression, cell death, tissue invasion/metastasis, and immune evasion. A striking feature of the results is that patients with cancers and many individuals not diagnosed with cancer could carry similar germline alterations in cell cycle, telomere maintenance, and DNA repair hallmark pathways (Fig. 3A,Additional file 2: Fig. S9C). Heatmaps of the CanSys scores that quantify the extent of alterations in the significantly altered cancer hallmark pathways in TCGA are also shown in Fig. 3B. CanSys quantifies pathway-level disturbances, which is not fully reflected in mutation frequencies in individual genes. For instance, *TP53* is one of the most frequently somatically mutated genes in cancer, found in 29.8% of TCGA samples, yet CanSys identified significant p53 pathway alterations (BH adjusted *P* < 0.25) in only 20.2% of samples due to somatic mutations, which is lower than the alteration frequencies in several other KEGG pathways (Additional file 2: Fig. S8A). This is consistent with preclinical and clinical observations that despite its powerful biological role, p53 mutations alone are not sufficient for malignant transformation [32, 33]. Pathway-level alterations depend on the contribution of many genes by integrating gene-level impact scores (Additional file 2: Fig. S10).

Different genes in the same pathway were affected in different individuals indicating phenotypic convergence. Additional file 1: Tables S5 and S6 and Additional file 2: Fig. S11 illustrate this for the GO and KEGG cell cycle pathways that consist of 1,252 and 157 genes, respectively. In pan-cancer analysis, germline alterations were broadly distributed across the genes without clear pattern by cancer type, whereas somatic alterations showed a clear cancer type-specific pattern. The Z-scores for phenotypic convergence were 7.22 and 5.41 for somatic and germline variants, respectively, for the GO cell cycle pathway. The corresponding Z-scores for the KEGG cell cycle pathway were 15.70 and 4.95. All empirical *P*-values remained below 0.05.

### Breast and lung cancer as case studies: pathway-level disturbances within different cancer types

**Fig. 4:**
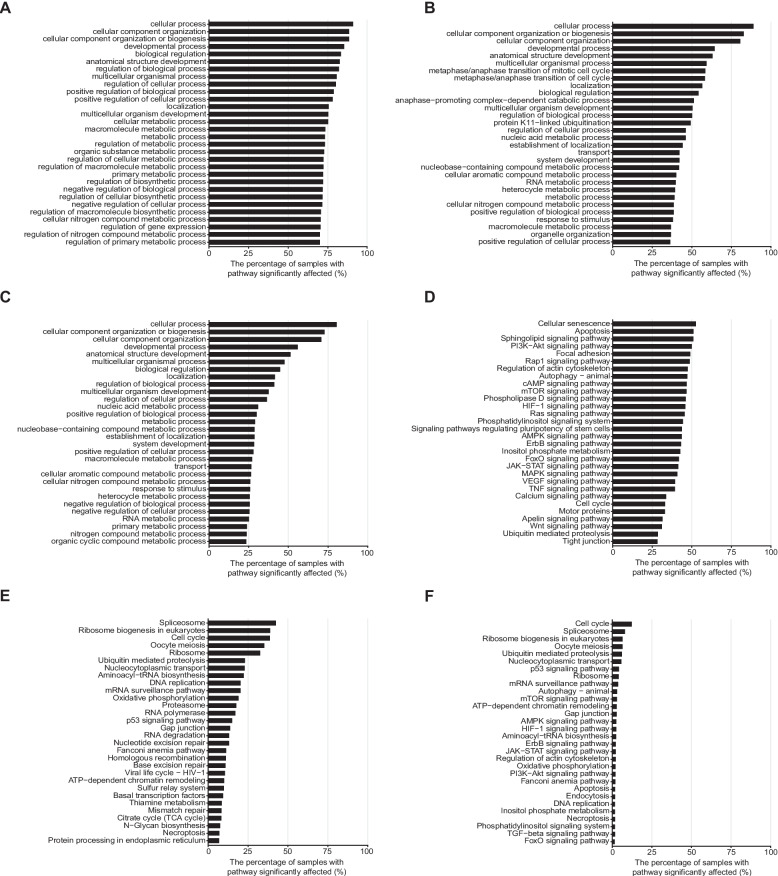
Significantly affected pathways in TCGA breast cancer samples. **A-C** Top 30 most frequently affected GO pathways categorized by the origin of alterations as somatic (**A**), germline (**B**), or both (**C**), with BH adjusted *P* < 0.25. **D-F** Top 30 most frequently affected KEGG pathways categorized origin of alterations as somatic (**D**), germline (**E**), or both (**F**), with BH adjusted *P* < 0.25. Germline variants were excluded if they had a gnomAD population allele frequency > 0.001 or a CADD score < 15. BH: Benjamini–Hochberg procedure

To further explore how these pathway-level disturbances manifest within a single cancer type, we selected breast cancer as a primary case study due to its pronounced clinical heterogeneity and well-characterized molecular subtypes. In TCGA breast cancer samples, the median number of somatic mutation affected GO pathways (BH adjusted *P* < 0.25) per cancer were 972 (range: 0–1,846), and 107 pathways (range: 0–1,764) were affected by germline variants, 28 (range: 0–336) were affected by both types of alterations. For KEGG pathways, the corresponding numbers were 26 (range: 0–65), 6 (range: 0–51), and 1 (range: 0–16). The overall pattern of results was similar to the pan-cancer analysis (Additional file 1: Table S7 and Table S8). The most frequently somatically altered pathways were cellular senescence, apoptosis and a broad range of signaling pathways encountered in 31–53% of cancers (Fig. 4A and D). A substantial subset of breast cancer patients had high functional impact germline variants in cell cycle, mitosis regulation, RNA processing, DNA replication, and DNA repair pathways (Fig. 4B and E). Similar analysis on the BEAUTY dataset yielded essentially identical results indicating robustness of the findings (Additional file 2: Fig. S12). Correlations for the percentages of samples with significantly affected (BH adjusted *P* < 0.25) GO and KEGG pathways by somatic or germline origins ranged from 0.6279 to 0.9680, and the pathways showed similar alteration frequencies in both cohorts. Average CanSys scores were also highly concordant, with correlations ranging from 0.5753 to 0.9424. All *P*-values remained below 0.05. Gene-level alterations in GO cell cycle and DNA repair, and KEGG cell cycle and nucleotide excision repair pathways in individual samples are shown on Additional file 2: Fig. S13 to illustrate that different pathway members were affected in different individuals.

Overall, 5,193 GO and 120 KEGG pathways were affected in > 5% of the TCGA breast cancer samples. Fewer pathways were affected by germline variants than by somatic mutations, but the germline affected pathways had greater consistency across samples (Additional file 2: Fig. S14). Heatmaps of the CanSys scores of significantly affected GO and KEGG pathways in > 5% of breast cancer samples are shown on Additional file 2: Fig. S15. Even when the same pathway is altered in many cancers, the magnitude of pathway-level disturbance varies from cancer-to-cancer. A total of 995 GO and 33 KEGG pathways were differentially affected between ER-positive and -negative cancers at the somatic level (BH adjusted Fisher’s exact test *P* < 0.05) (Additional file 1: Table S9 and Table S10, Additional file 2: Fig. S16). The ER-positive cancers more frequently harbored somatic mutations in signal transduction pathways (TNF, Ras, VEGF, Rap1, cAMP, FoxO, ErbB, AMPK, JAK-STAT, mTOR, HIF-1, PI3K-Akt signaling pathways, and Phospholipase/Phosphatidylinositol/Inositol phosphate signaling pathways), whereas ER-negative cancers more frequently had somatic mutations in ferroptosis, mitophagy, WNT, and p53 pathways. Compared with CanSys, DAVID identified fewer differently affected pathways by somatic mutations between the two groups (Additional file 2: Fig. S17A and B). DAVID identified seven GO pathways (gland development, regulation of cell projection organization, regulation of cell–cell adhesion, regulation of plasma membrane bounded cell projection organization, regulation of cell adhesion, cell migration and cell motility) and one KEGG pathway (Rap1 signaling pathway), all of which were also identified by CanSys.

**Fig. 5:**
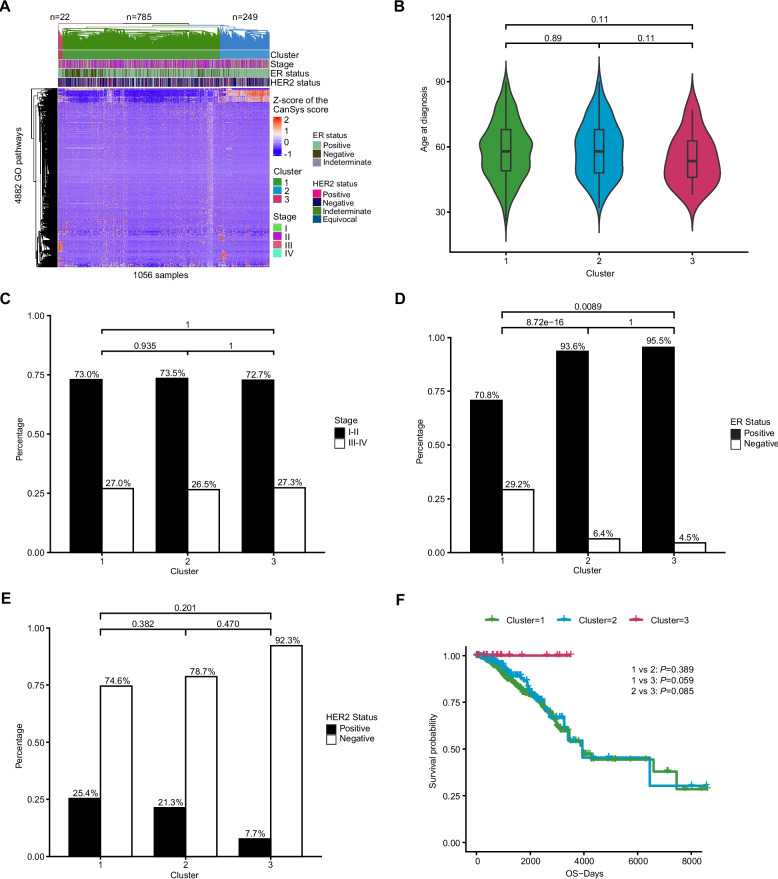
Hierarchical clustering based on CanSys scores from somatic-GO results in TCGA breast cancer samples. **A** Heatmap of the z-scores of CanSys scores showing distinct patterns across clusters. Comparisons of age at diagnosis (**B**), AJCC stage (**C**), Estrogen Receptor (ER) status (**D**), HER2 status (**E**), and overall survival (**F**) across the three clusters. The Z-scores were capped at a range from −2 to 2. Statistical analyses were performed using the Wilcoxon rank-sum test for age at diagnosis, Fisher’s exact test for stage, ER statuses, and HER2 statuses, and the log-rank test for survival analysis

Hierarchical clustering of CanSys scores revealed 3 clusters comprising 785, 249, and 22 samples, respectively (Fig. 5,Additional file 1: Table S11). The three clusters had distinct patterns of somatic alterations in GO pathways (Additional file 1: Table S12). There was no difference in stage distribution between the clusters, but cluster 3 had slightly younger patients and included the highest proportion of ER-positive (96%) and the lowest proportion of HER2-positive (6.7%) cancer. Cluster 3 also had an unusually good survival (100%). This group was characterized by pathway alterations in sphingosine-1-phosphate receptor signaling [34, 35] and nitric oxide biosynthetic process [36, 37], along with numerous immune and metabolic pathways.

In addition to breast cancer, we compared pathway-level alterations between LUAD and LUSC in TCGA lung cancer samples identified by CanSys and DAVID, respectively. Overall, CanSys identified a substantially larger number of significantly differently affected pathways than DAVID (Additional file 1: Table S13 and Table S14, Additional file 2: Fig. S17C and D). CanSys and DAVID yielded partially discordant results. For example, the KEGG p53 signaling and cellular senescence pathways were identified by DAVID as enriched in LUSC, whereas CanSys identified these pathways as having higher alteration prevalence in LUAD.

The robustness of the CanSys results was further supported by comparisons of LUAD samples from the TCGA and EAS-LUAD cohorts (Additional file 2: Fig. S18). Although the EAS-LUAD patients are all from East Asian population, in contrast to the mostly White TCGA cohort, the correlations for the percentages of samples with GO and KEGG pathways significantly affected (BH adjusted *P* < 0.25) in the somatic and germline analyses remained high, ranging from 0.5892 to 0.8457. Average CanSys scores were also concordant between cohorts, with correlations ranging from 0.6712 to 0.8676. All *P*-values remained below 0.05. The most frequently somatically altered pathways included cellular senescence and a broad range of signaling pathways (Ras, Rap1, mTOR, FoxO, MAPK, ErbB, and PI3K–Akt). A substantial proportion of LUAD samples in both cohorts carried high functional-impact germline variants in cell cycle, RNA processing, DNA replication, and DNA repair pathways.

## Discussion

Why one in three individuals develops cancer during their lifetime in the United States, and why cancers with identical histologic features have distinct clinical courses are poorly understood. The large patient-to-patient variation in the clinical behaviors of similar stage cancers resembles the large variations in our personal features. Our biological differences are attributed to a combination of inherited genetic and acquired environmental factors. In this study, we examined the contribution of germline polymorphisms and somatic mutations to alterations in biological processes that are implicated in cancer biology and assessed the patterns of pathway alterations across cancer types. We developed a new tool to quantify the effect of gene alterations from germline and somatic origins on biological pathways and constructed two network spaces to visualize these alterations in the standard relational framework of the GO and KEGG databases. To quantify pathway affectedness, we first calculated a gene-level functional impact score that combines the functional importance of a gene with the predicted impact of the germline variants and somatic mutations that it harbors, then calculated a pathway-level gene alteration enrichment score to identify significantly affected pathways. Finally, we calculated a pathway-level alteration score, termed the CanSys score, by integrating enrichment scores with the sum of gene impact scores.

By applying our method to TCGA data, we found that a very large number of biological processes are altered with variable frequencies and to various extents across cancers. Somatic mutations affect a substantially larger number of pathways across a greater proportion of samples than germline variants. The most surprising finding of our analysis is that DNA repair and cell cycle were frequently affected at the germline level, regardless of cancer type. Frequent alterations in these pathways were also observed in individuals without cancer in the 1kGP cohort, indicating that functional germline variants capable of perturbing these biological pathways are present across the general human population. The variants that caused these pathway alterations were polymorphisms with high predicted functional impact but without known association with cancer risk in GWAS studies suggesting that their effect is genomic context dependent. Furthermore, different genes and combinations of genes were affected in different individuals that we interpret as phenotypic convergence. These results suggest that subtle inherited forms of dysregulation in cell cycle and DNA repair are present in a large fraction of the population, and these could contribute to cancer risk by lowering the threshold for malignant transformation.

Somatic alterations primarily affected genes involved in metabolism, cytoskeleton, cell adhesion, and signal transduction processes and showed greater variability across cancers. Cellular process, cellular component, development, regulation, metabolism and cell differentiation were the most frequently affected GO pathways by somatic mutations across cancer types. This is consistent with the concept of cancer hallmarks which implies that all cancers share biological similarities that enable invasion, metabolic rewiring, and escape from physiologic regulation of cell proliferation, apoptosis, and immune surveillance. In addition to the broadly shared pathway alterations, every cancer also had many rare pathway alterations in unique combinations. We hypothesize that these sample-specific alterations could influence the individual biological behavior of cancers. We also observed that certain genes are preferentially affected by somatic mutations whereas others are frequently affected by both somatic mutations and germline variants; these patterns vary by cancer type.

In breast cancer samples, cellular and metabolic processes, and DNA damage repair and cell cycle pathways, were the most frequently affected GO pathways at both somatic and germline levels. The cell cycle pathway was the most commonly affected KEGG pathway at both somatic and germline levels, whereas apoptosis and signaling pathways of PI3K-Akt, mTOR, ErbB, and Wnt showed the most frequent somatic-level disturbances. We found large differences in pathway alterations between ER-positive and ER-negative samples from TCGA, consistent with the known differences in the biology of these different breast cancer subtypes. For example, mTOR and PI3K-Akt signaling pathways were more frequently altered in ER-positive samples [38], whereas WNT and p53 pathways showed higher alteration frequencies in ER-negative samples [39, 40]. Furthermore, CanSys identified substantially more significantly differentially affected pathways than DAVID. The difference was also observed in the comparison between LUAD and LUSC in TCGA lung cancer samples. CanSys and DAVID yielded partially discordant results. These discrepancies indicate that incorporating variant deleteriousness and gene-level importance leads to pathway-level results that differ from those obtained by gene-count–based enrichment analyses, and could better reflect functional differences in pathway activity.

Our study has limitations. To quantify the biological importance of a gene we used DepMap scores that reflect a gene’s essentiality to sustaining cell survival in vitro, this metric does not capture other biological functions that are also important for cancer biology. No genome-wide DepMap-like gene annotations exist for other phenotypic effects such as invasiveness or immune evasion. A study using CRISPR screens in 3D cultures (spheroids) have suggested an improved way of developing physiologically more accurate measures of cancer driver potential for genes [41]. The DepMap consortium is also expanding to include 3D patient-derived models, we recognize this as an important future opportunity to improve the CanSys model. We assumed that germline polymorphism with CADD score ≥ 15 alter protein function in a way that it affects the biological process that a gene is involved with, this assumption is based on algorithmic predictions to estimate the impact of DNA sequence alterations on protein function but few of these are experimentally validated. Experimental assessment of functional impact of millions of germline variants is beyond current feasibility. Other deleteriousness metrics, such as AlphaMissense and VARITY, have the potential to replace CADD; however, their current versions have limited genomic coverage. Future versions of CanSys could incorporate these tools once more comprehensive precomputed datasets become available. Integrating multi-omics data can provide a more complete view of biological systems, as demonstrated by other computational tools such as PARADIGM [42], ONCOIMPACT [43] and SCS [44]. Our current method does not incorporate copy number and structural variants, or epigenetic changes that also affect biological pathway integrity, due to the lack of methods that integrate multi-omics data while explicitly modeling variant deleteriousness and gene importance. All pathway analysis methods are limited by lack of ability to integrate the directional impact of gene-level alterations and accurately infer if the net effect is an increased or decreased pathway output. Despite these limitations, CanSys is one of the few tools that not only identifies significantly disturbed pathways but also quantifies pathway disturbances at single-sample level. This study used biological pathways from GO and KEGG, but it is possible to extend our web-based CanSys tool to support any other collection of gene sets. Previous pathway analysis tools, including DAVID, Reactome [45], traditional or single sample GSEA [46], Ingenuity Pathways Analysis (IPA), STAGEs [47], GeneCloudOmics [48], GENAVi [49], iDEP [50] have been widely used to identify functionally enriched pathways in genomic or mRNA expression datasets. However, these tools primarily focus on detecting statistical enrichment across groups, rather than quantifying the extent of pathway perturbation in individual samples. Unlike DAVID and Reactome, which rely on predefined gene sets and overrepresentation analysis, in CanSys we integrate genetic alterations and assign a quantitative pathway alteration score, allowing a more nuanced interpretation of functional disruptions. Additionally, while traditional GSEA and IPA can assess pathway-level significance, they do not account for gene-specific functional importance that we derived from large-scale dependency datasets. CanSys also differs in its ability to visualize individual pathway disturbances interactively, providing us with sample-specific insights rather than population-wide trends. In the analysis of TCGA samples, we did not restrict samples based on population stratification to maximize sample size for each cancer type.

## Conclusions

This study introduces a novel method for mapping germline and somatic alterations onto GO and KEGG pathways and quantifying pathway-level disturbances in individual samples. Using this approach, we highlight consistent and biologically meaningful patterns across cancer types. Specifically, we show that a substantial proportion of cancers, regardless of cancer type, carry high functional impact germline variants in cell cycle regulation, telomere maintenance, and DNA repair pathways, whereas somatic mutations primarily affect pathways involved in cell adhesion, cell motility, metabolism, and a broad range of signal transduction processes. Pathway alterations are mediated by different genes in different cancers indicating phenotypic convergence, and the unique combination of pathway alterations might explain the unique behavior of each cancer.

## Supplementary Information

Additional file 1: Supplementary Tables. This file contains all supplementary tables.

Additional file 2: Supplementary Figures. This file contains all supplementary figures.

## Data Availability

The mutation and expression data from TCGA are available via the website (https://portal.gdc.cancer.gov/) [18]. The raw whole WES and RNA-seq data from the BEAUTY are available in the dbGaP database under accession number phs001050.v1.p1 (https://www.ncbi.nlm.nih.gov/projects/gap/cgi-bin/study.cgi?study_id=phs001050.v1.p1 [19]. The raw whole WES and RNA-seq data from the EAS-LUAD cohort are available in the EGA database under accession number EGAD00001004421 and EGAD00001004422 (https://ega-archive.org/dacs/EGAC00001002039) [20]. The source code for the CanSys tool, which also provides a command-line version for applying it to custom datasets, is available on GitHub (https://github.com/jwdelta/CanSys) [51] and Zenodo (https://zenodo.org/records/19225404) [52].

## Abbreviations

1kGP: The 1000 Genomes Project
AD: Allelic depth
BEAUTY: Breast Cancer Genome Guided Therapy Study
BFS: Breadth-first search
BH: Benjamini-Hochberg
BRCA: Breast cancer
BWA: Burrows–Wheeler Aligner
CADD: Combined Annotation Dependent Depletion
CHOL: Cholangiocarcinoma
DAVID: Database for Annotation, Visualization, and Integrated Discovery
DepMap: Cancer Dependency Map
EAS-LUAD: East Asian-ancestry Lung Adenocarcinoma
ER: Estrogen receptor
ES: Enrichment score
GATK: Genome Analysis Toolkit
GBM: Glioblastoma multiforme
gnomAD: Genome Aggregation Database
GO: Gene Ontology
GSEA: Gene Set Enrichment Analysis
HER2: Human epidermal growth factor receptor 2
IHC: Immunohistochemistry
INDELs: Insertion/deletions
IPA: Ingenuity Pathways Analysis
KEGG: Kyoto Encyclopedia of Genes and Genomes
KICH: Kidney chromophobe
KIRC: Kidney renal clear cell carcinoma
LAML: Acute myeloid leukemia
LGG: Brain lower grade glioma
LUAD: Lung adenocarcinoma
LUSC: Lung squamous cell carcinoma
MESO: Mesothelioma
MVC: Model-View-Controller
PAAD: Pancreatic adenocarcinoma
PRAD: Prostate adenocarcinoma
SNVs: Single nucleotide variants
SRA: Sequence Read Archive
STAR: Spliced Transcripts Alignment to a Reference
TCGA: The Cancer Genome Atlas
TGCT: Testicular germ cell tumors
THCA: Thyroid carcinoma
UCS: Uterine carcinosarcoma
UVM: Uveal melanoma
VAF: Variant allele frequency
VCF: Variant Call Format
WES: Whole exome sequencing
