# Supplementary material for: Functional germline variants together with somatic mutations alter the integrity of cancer hallmark regulatory networks
Source: Genome Med. 2026 Apr 29;18:59. doi: 10.1186/s13073-026-01644-8 (PMC13151107; doi:10.1186/s13073-026-01644-8)
Supplement: Supplementary file 2 — Additional file 2: Supplementary Figures. This file contains all supplementary figures. [file 13073_2026_1644_MOESM2_ESM.docx]

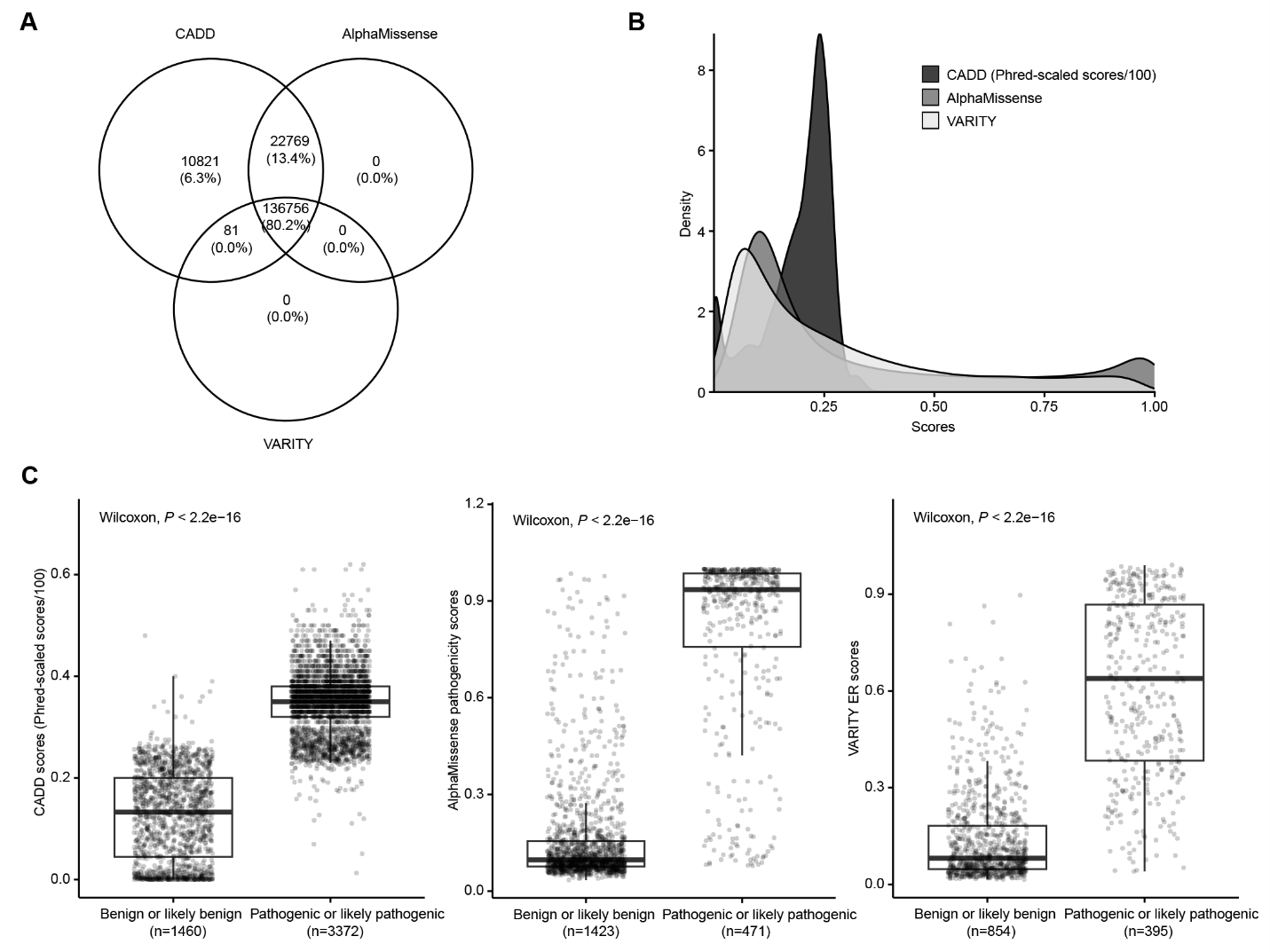


**Fig. S1: Comparison of annotation overlap and deleteriousness predictions among CADD, AlphaMissense, and VARITY for all possible nonsynonymous SNVs (n=170,427) from ten frequently mutated DNA repair genes (*ATM*, *BRCA2*, *PRKDC*, *ATR*, *BRCA1*, *MSH6*, *POLE*, *FANCM*, *FANCD2*, and *SLX4*).** **A** Overlap of annotations for 170,427 nonsynonymous SNVs from ten frequently mutated DNA repair genes. **B** Density distributions of deleteriousness scores for the 126,756 variants jointly annotated by CADD, AlphaMissense, and VARITY. **C** Comparison of deleteriousness scores between benign/likely benign and pathogenic/likely pathogenic variants across CADD, AlphaMissense, and VARITY, using ClinVar annotations as a reference. Statistical significance was assessed using the Wilcoxon rank-sum test.

**
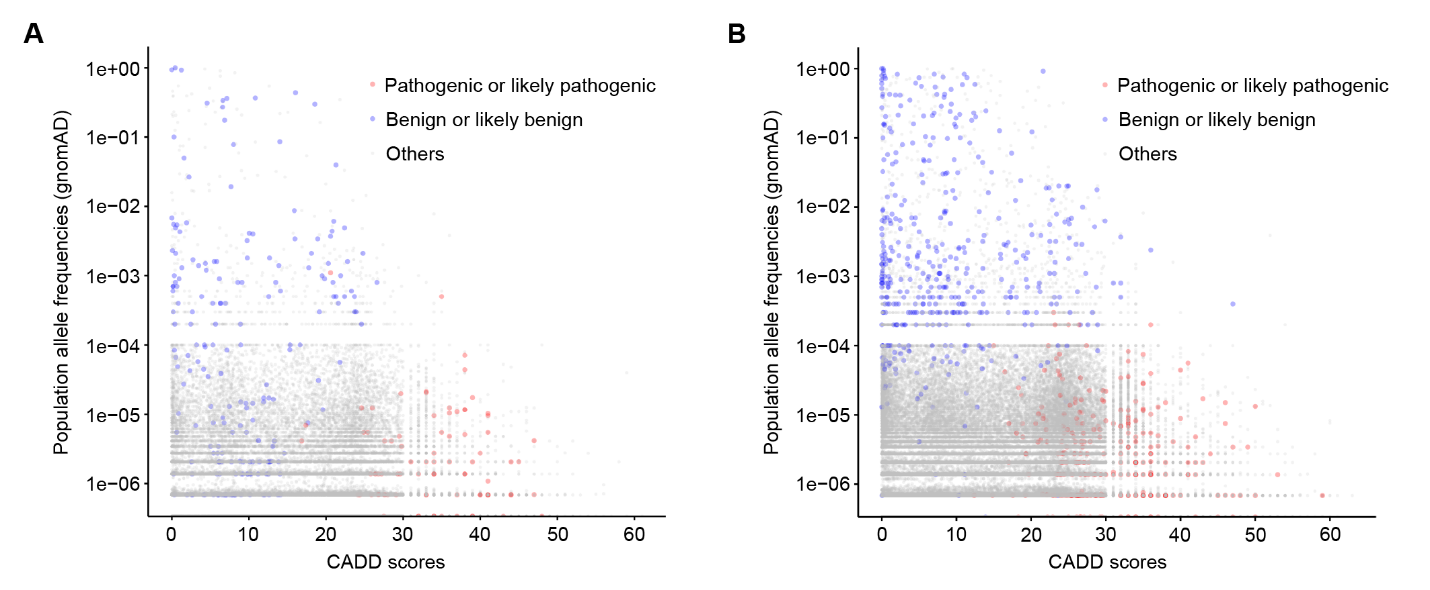
**

**Fig. S2: CADD scores and population allele frequencies of all annotatable variants located in the exonic regions of two cancer-associated chromosomal bands.** **A** Variants in the exonic regions of chromosomal band 10q23 (n=35,336). **B** Variants in the exonic regions of chromosomal band 17p13 (n=106,355). Variants annotated in ClinVar as pathogenic or likely pathogenic and benign or likely benign are shown in red and blue, respectively. Other variants are shown in gray with smaller point size.


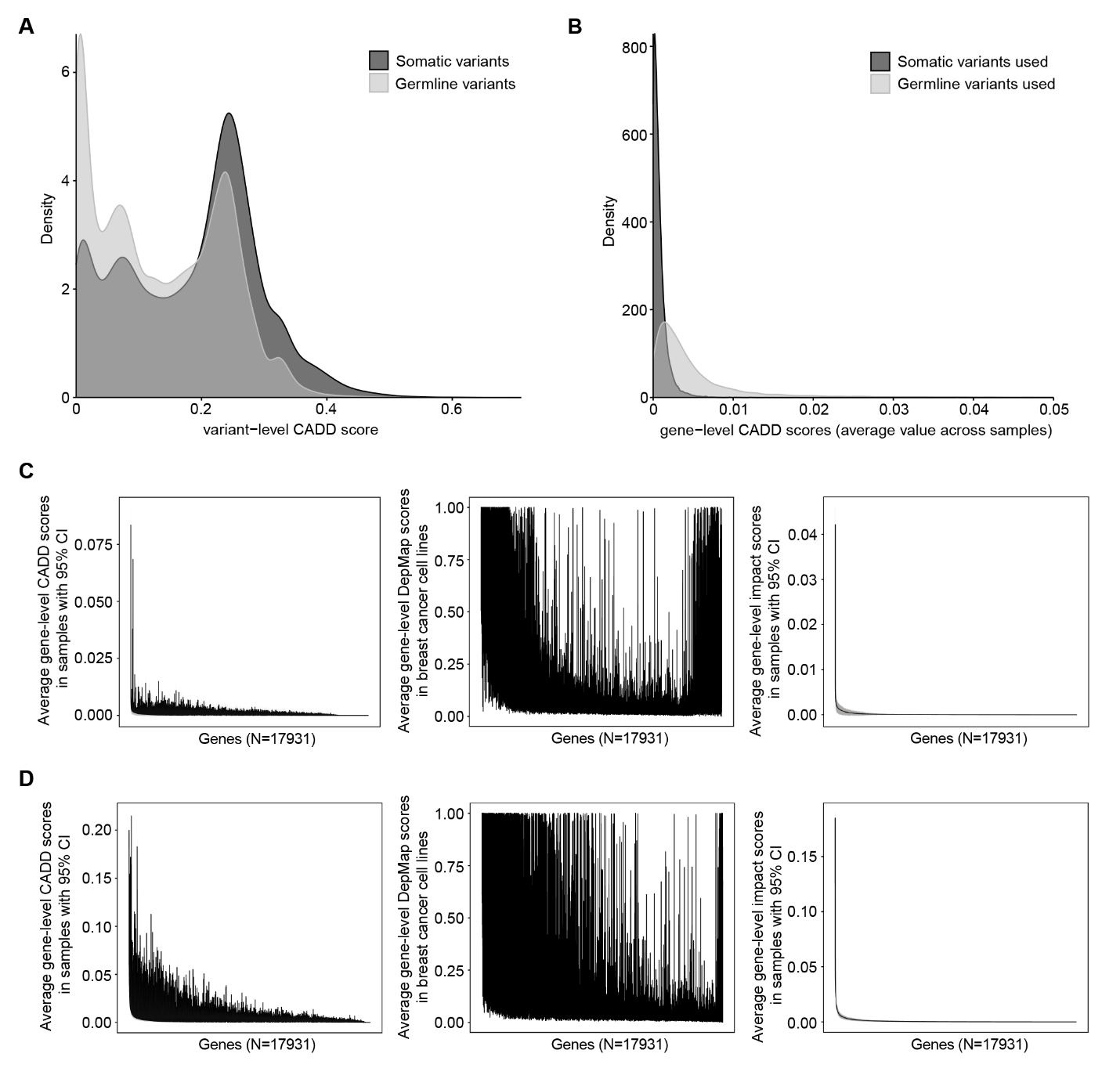


**Fig. S3: Overview of CADD scores, DepMap scores and gene-level impact scores in TCGA breast cancer samples. A** The distribution of variant-level CADD scores. **B** The distribution of average gene-level CADD scores across samples. **C** The contribution of CADD scores and DepMap scores to gene-level impact scores, using data from somatic variants, with identical gene ordering of 17,931 genes. **D** The contribution of CADD scores and DepMap scores to gene-level impact scores, using data from germline variants, with identical gene ordering. Germline variants were excluded if they had a gnomAD population allele frequency > 0.001. No threshold for CADD scores was used in this analysis.


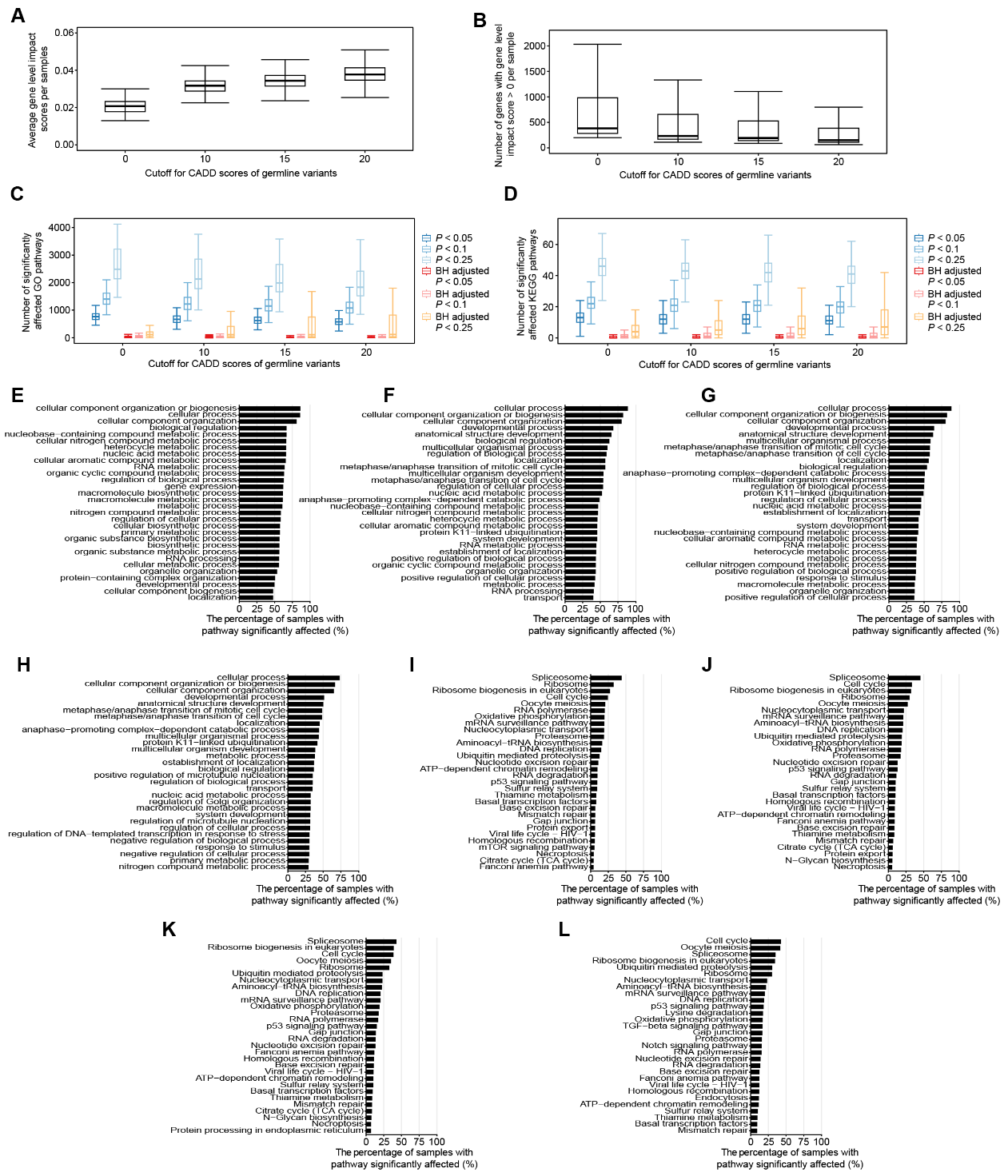


**Fig. S4: The impact of selecting different germline variant CADD score thresholds on CanSys results in TCGA breast samples.** **A** Using a higher cutoff to filter germline variants results in increased average gene-level impact scores. **B** Using a higher cutoff to filter germline variants results in decreased number of genes with gene-level impact scores > 0. **C, D** The number of significantly affected GO and KEGG pathways at the germline level, across various CADD score cut off values and significance thresholds. **E-H** Top 30 most frequently affected GO pathways at the germline level, with BH adjusted *P*-values < 0.25, for CADD score cutoffs 0, 10, 15, and 20. **I-L** Top 30 most frequently affected KEGG pathways at the germline level, with BH adjusted *P*-values < 0.25, for CADD score cutoffs 0, 10, 15, and 20. Germline variants were excluded if they had a gnomAD population allele frequency > 0.001. BH: Benjamini-Hochberg procedure to adjust for multiple comparisons.


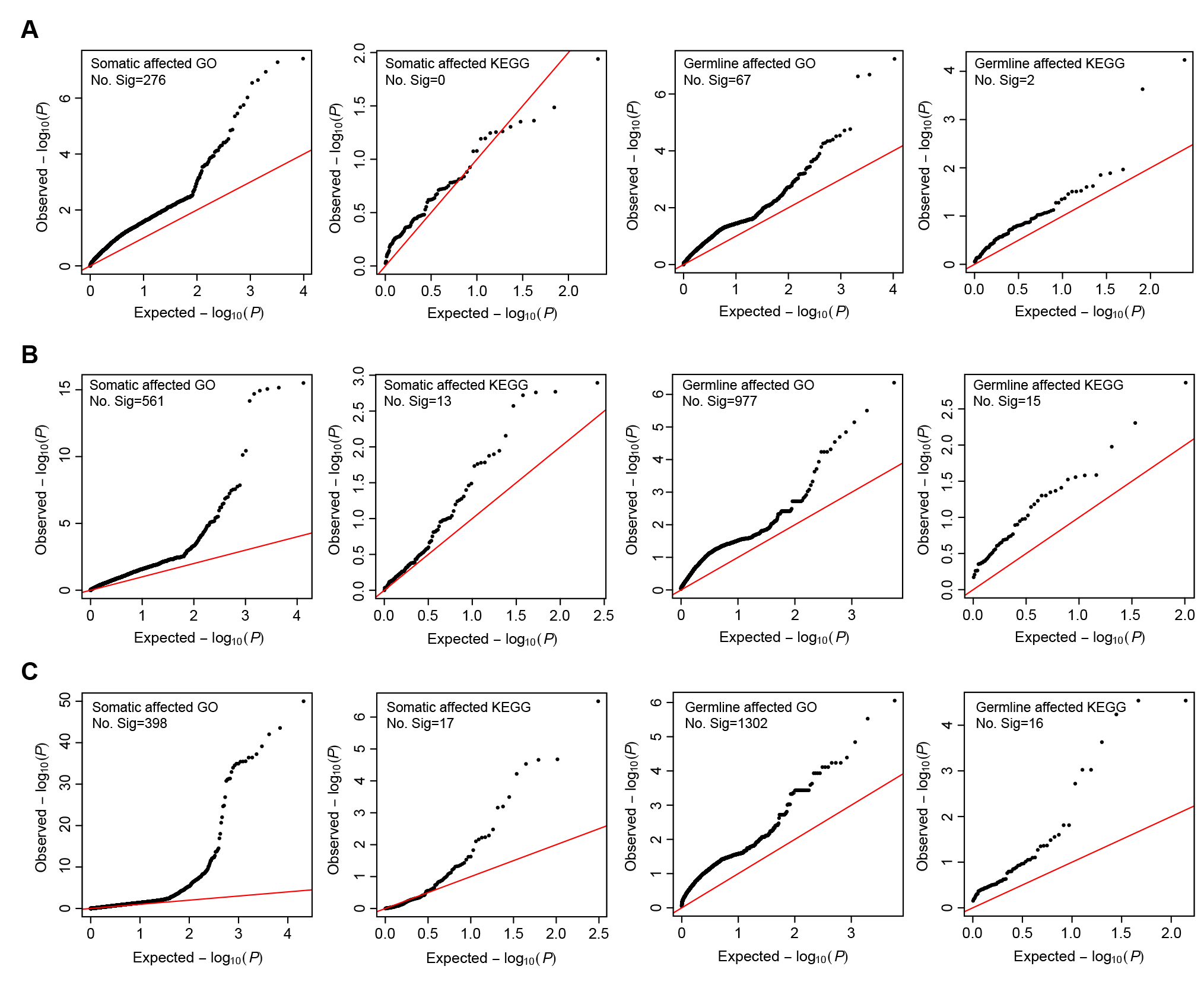


**Fig. S5: QQ plots of** **three randomly selected TCGA samples with varying numbers of significantly affected pathways.** **A** QQ plot for sample TCGA-3C-AALI-01A. **B** QQ plot for sample TCGA-5L-AAT1-01A. **C** QQ plot for sample TCGA-AC-A23H-01A. Each panel is subdivided into four QQ plots, showing the results corresponding to the GO and KEGG analyses of somatic mutations and germline variants, respectively. Germline variants were excluded if they had a gnomAD population allele frequency > 0.001 or a CADD score < 15. The number of significantly affected pathways (BH adjusted *P* < 0.25) is labeled for each analysis. BH: Benjamini-Hochberg procedure to adjust for multiple comparisons.


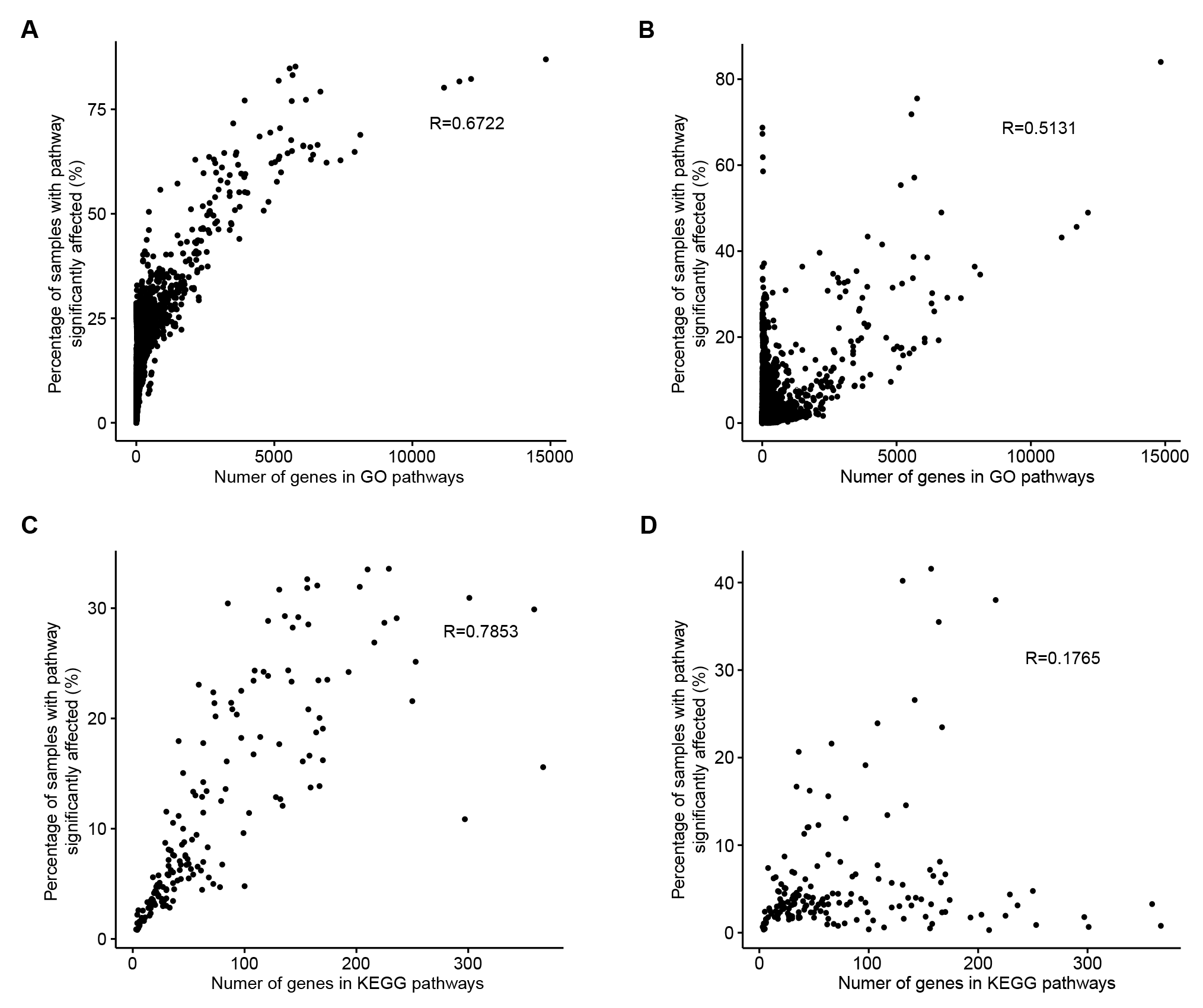


**Fig. S6: Pathway size and percentage of samples with pathway significantly affected across TCGA samples. A,B** Relationship between pathway size and the percentage of samples in which each GO pathway is significantly affected in somatic and germline analyses, respectively. **C,D** Relationship between pathway size and the percentage of samples in which each KEGG pathway is significantly affected in somatic and germline analyses, respectively. Germline variants were excluded if they had a gnomAD population allele frequency > 0.001 or a CADD score < 15. R represents the Pearson correlation coefficient computed across pathways.


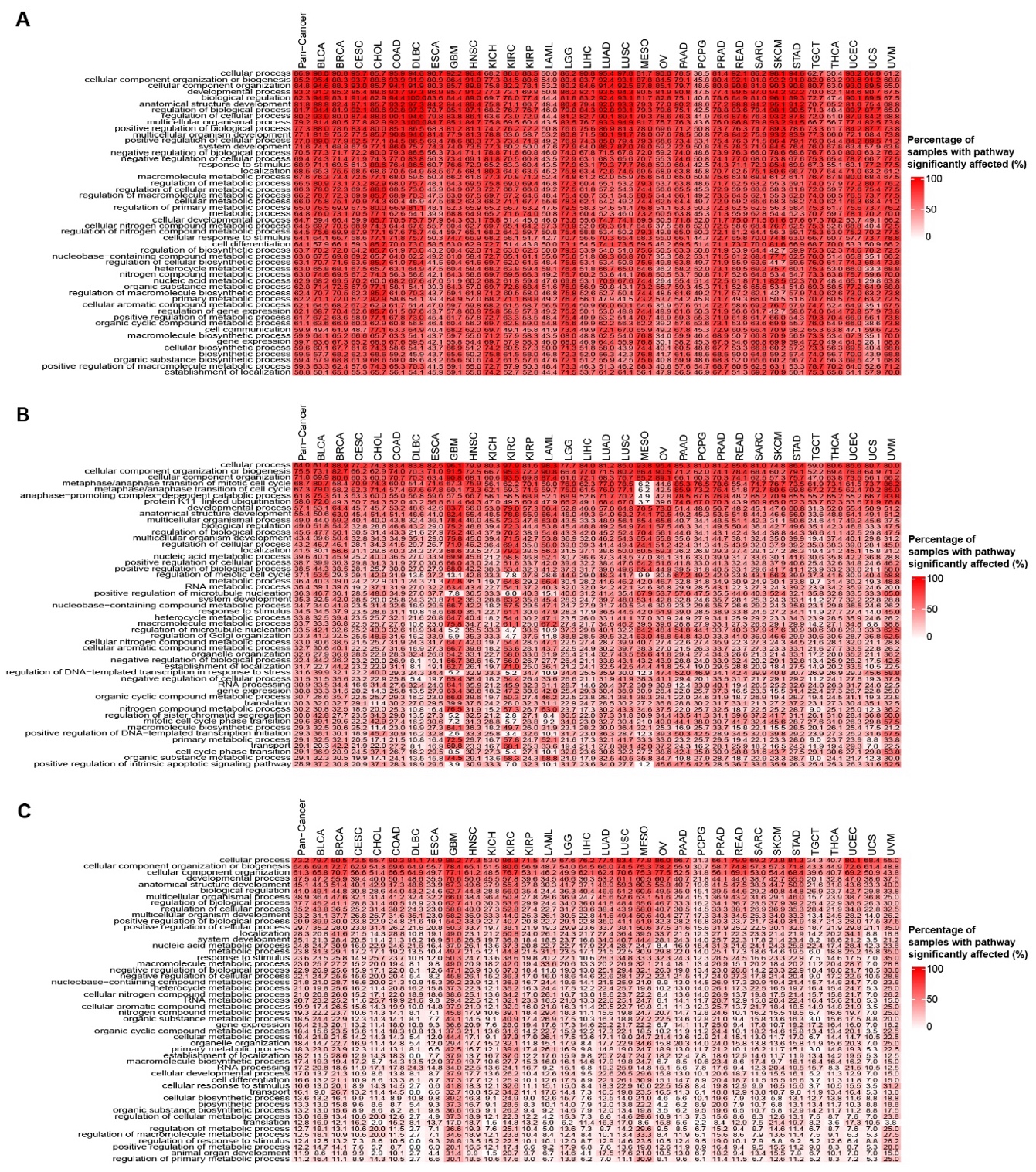


**Fig. S7: The top 50 most frequently significantly affected (BH adjusted *P* < 0.25) GO pathways across all cancer types in TCGA. A** The numbers in the heat map indicate the percentage of samples of a given cancer type that has pathway alteration due to somatic mutation. Pathways are listed on the Y-axis. Results are shown for the top 50 most commonly somatically altered GO terms. **B** The percentage of samples with pathway alteration at the germline level in the top 50 most commonly germline altered GO terms. **C** The percentage of samples with pathways overlapping affected by both somatic and germline alterations in the top 50 most commonly altered GO terms. Germline variants were excluded if they had a gnomAD population allele frequency > 0.001 or a CADD score < 15. BH: Benjamini-Hochberg procedure to adjust for multiple comparisons.


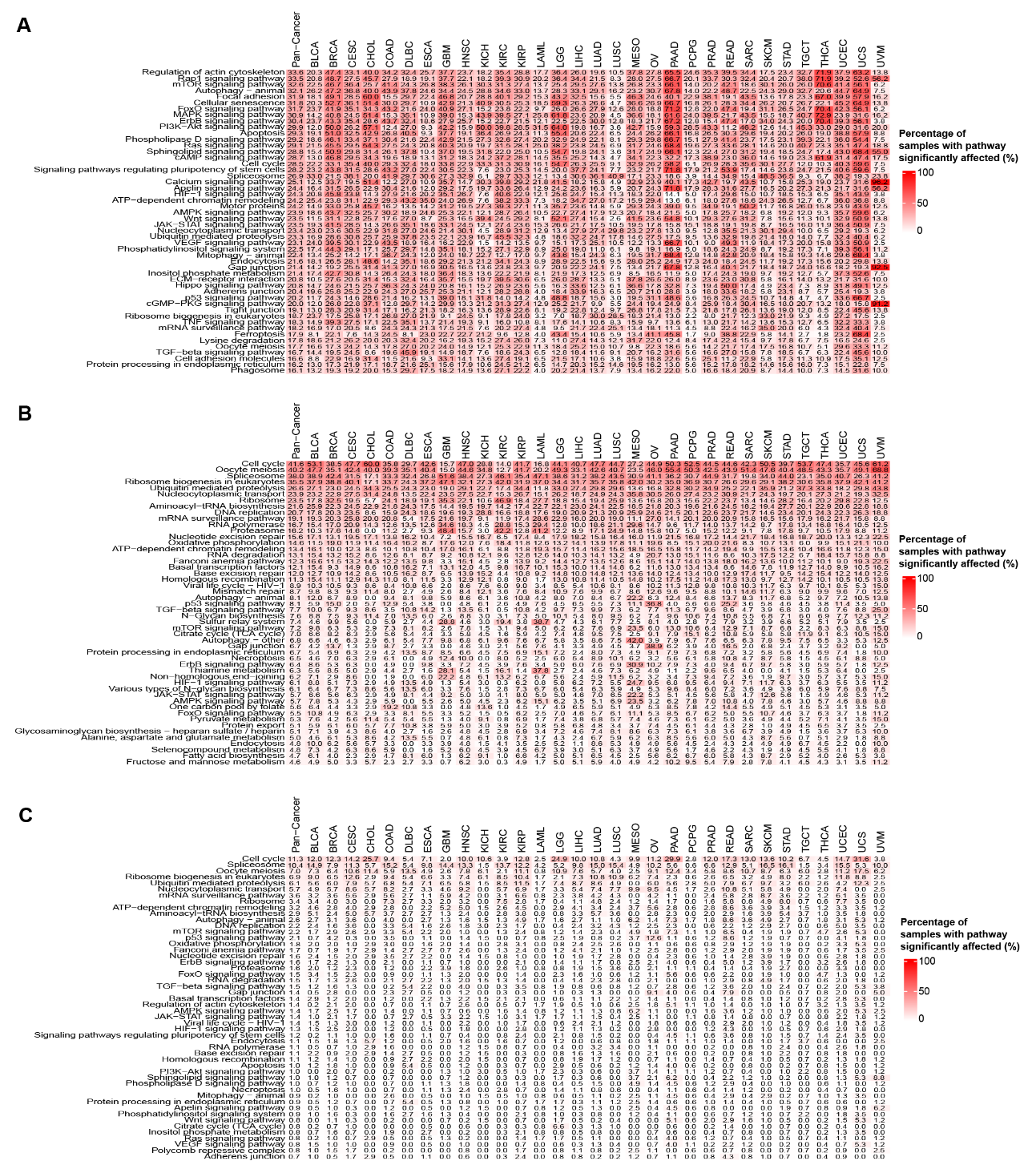


**Fig. S8: The top 50 most frequently significantly affected (BH adjusted *P* < 0.25) KEGG pathways across all cancer types in TCGA. A** The numbers in the heat map indicate the percentage of samples of a given cancer type that has pathway alteration due to somatic mutation. Pathways are listed on the Y-axis. Results are shown for the top 50 most commonly somatically altered KEGG pathways. **B** The percentage of samples with pathway alteration at the germline level in the top 50 most commonly germline altered KEGG pathways. **C** The percentage of samples with pathways overlapping affected by both somatic and germline alterations in the top 50 most commonly altered KEGG pathways. Germline variants were excluded if they had a gnomAD population allele frequency > 0.001 or a CADD score < 15. BH: Benjamini-Hochberg procedure to adjust for multiple comparisons.

**
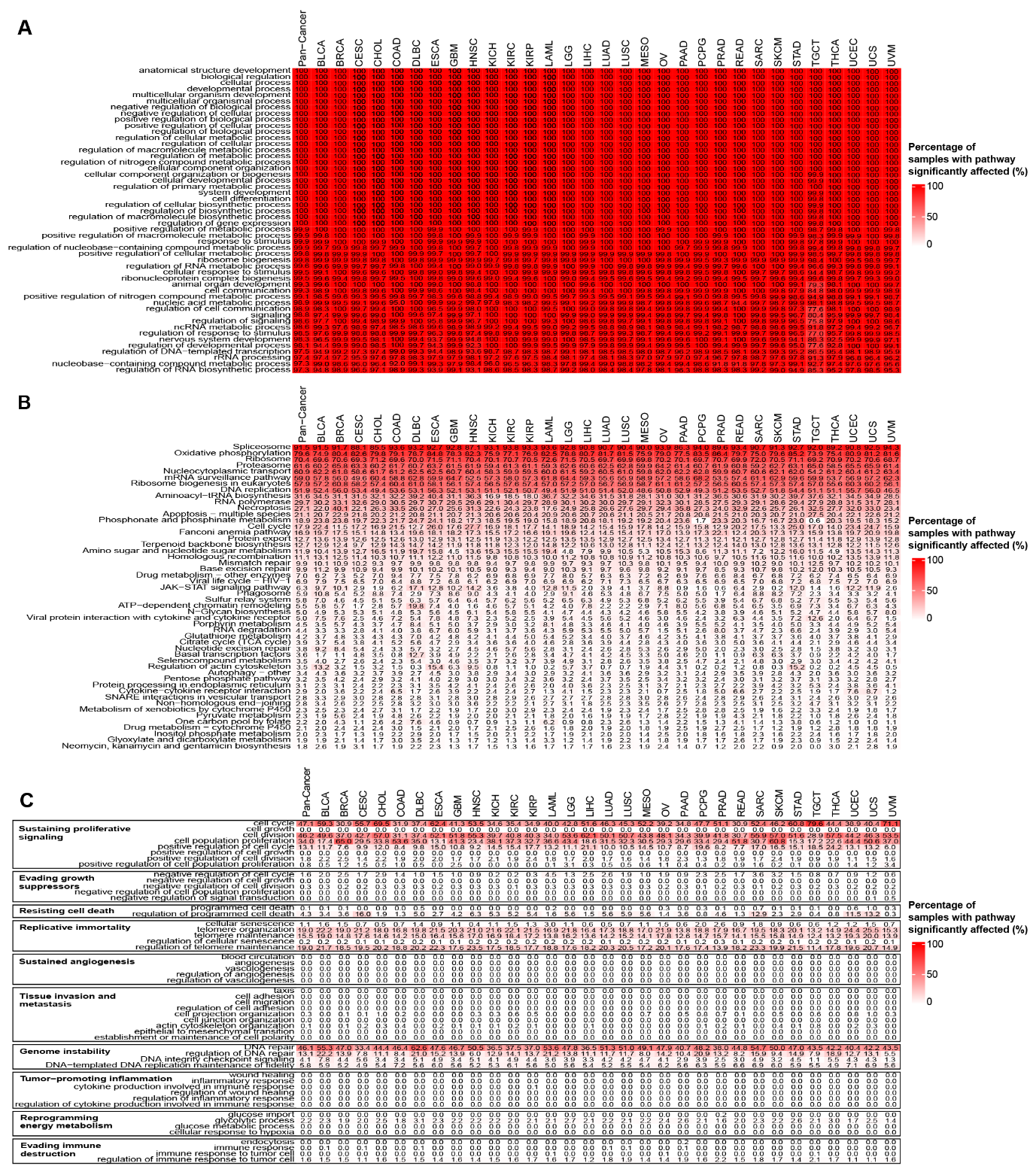
**

**Fig. S9: Pathway-level disturbances in the 1kGP cohort, generated using the same cancer-type–specific DepMap weights and expression-based filtering criteria as applied in the TCGA cancer cohorts. A** The top 50 most frequently significantly affected GO pathways. **B** The top 50 most frequently significantly affected KEGG pathways. **C** Alteration prevalence of the 52 GO terms corresponding to the 10 cancer hallmarks. Germline variants were excluded if they had a gnomAD population allele frequency > 0.001 or a CADD score < 15. The numbers in the heatmaps indicate the percentage of samples that has pathway alteration (BH adjusted *P* < 0.25). BH: Benjamini-Hochberg procedure to adjust for multiple comparisons.


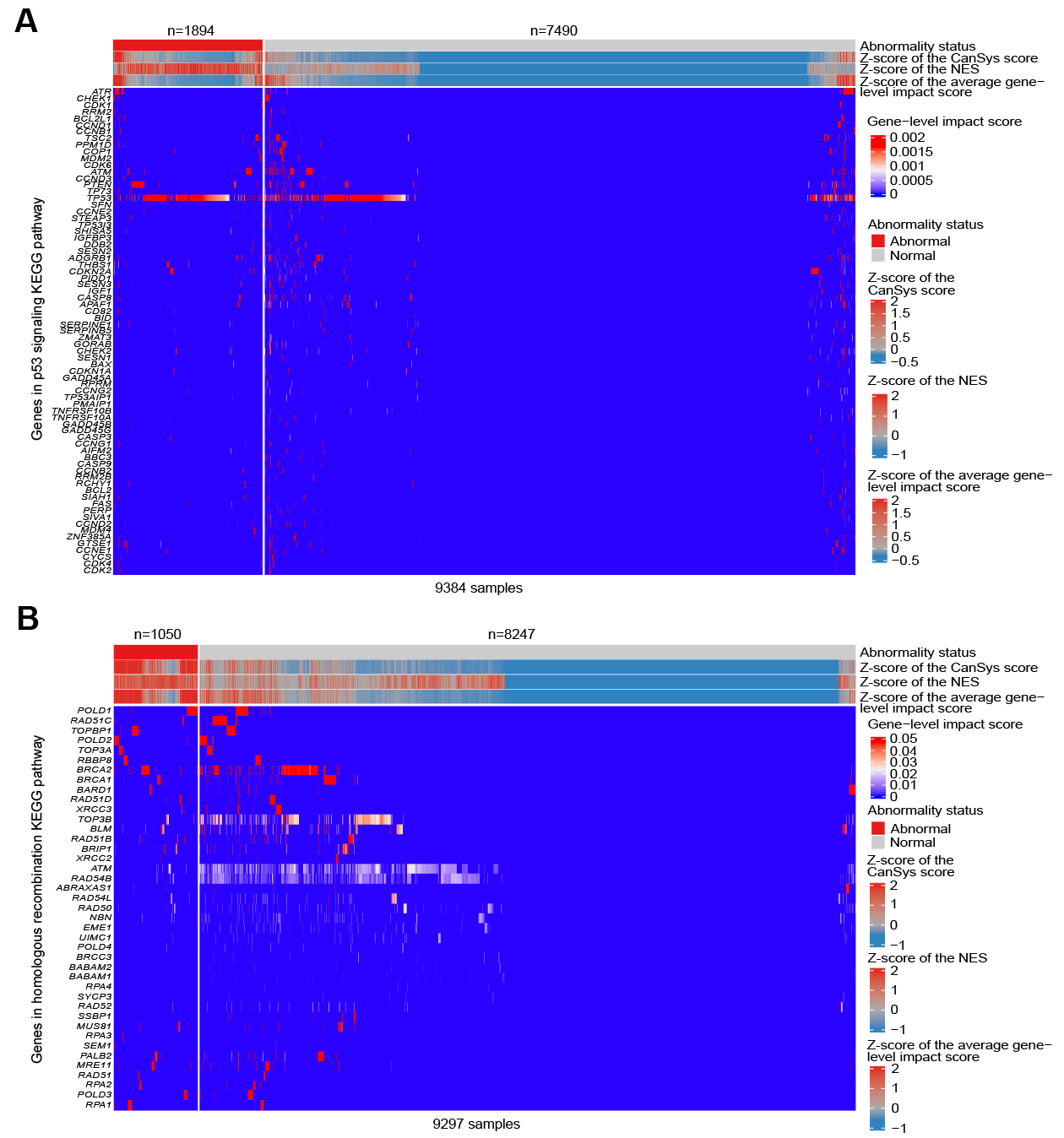


**Fig. S10: Heatmap of gene-level impact scores and corresponding z-scores for NES and the CanSys Scores for two representative KEGG pathways in TCGA samples.** Samples were grouped by pathway abnormality status defined as statistically significant altered (BH adjusted *P* < 0.25, i.e. abnormal), or not. **A** 74 genes in the p53 signaling pathway in the KEGG affected by somatic variants. **B** 41 genes in the homologous recombination pathway in the KEGG affected by germline variants. Germline variants were excluded if they had a gnomAD population allele frequency > 0.001 or a CADD score < 15. NES: normalized enrichment score. BH: Benjamini-Hochberg procedure.


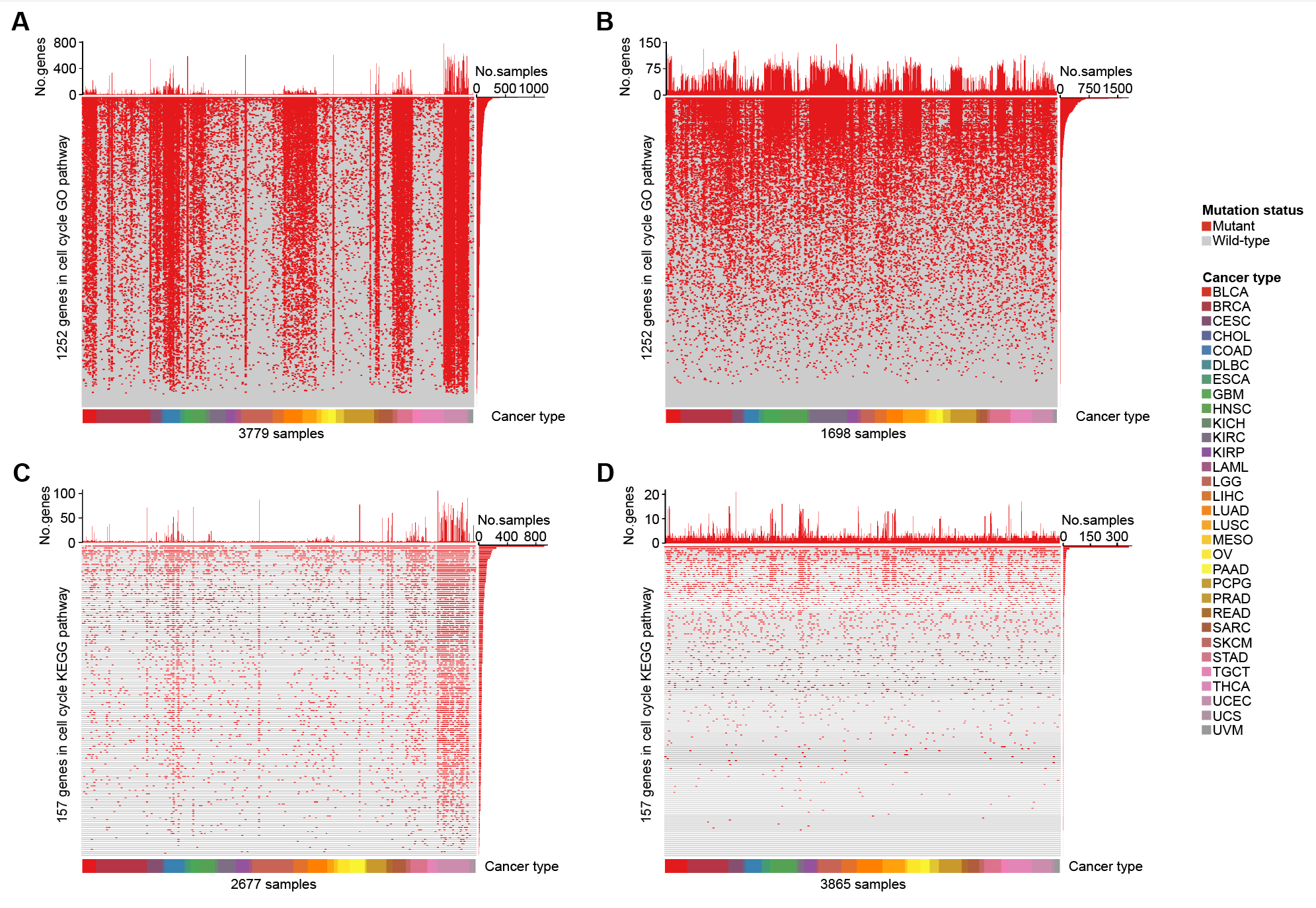


**Fig. S11: Oncoplot of gene-level alterations in GO and KEGG cell cycle pathway genes in TCGA.** Only samples with significant cell cycle pathway alteration (BH adjusted *P* < 0.25) are included in the figure. Mutation status of 1,252 genes included in the GO cell cycle term affected by somatic **(A**) or germline variants (**B**). Mutation status of 157 genes that comprise the KEGG cell cycle pathway, affected by somatic (**C**) and germline variants (**D**). Germline variants were excluded if they had a gnomAD population allele frequency > 0.001 or a CADD score < 15. Samples aligned by cancer type.


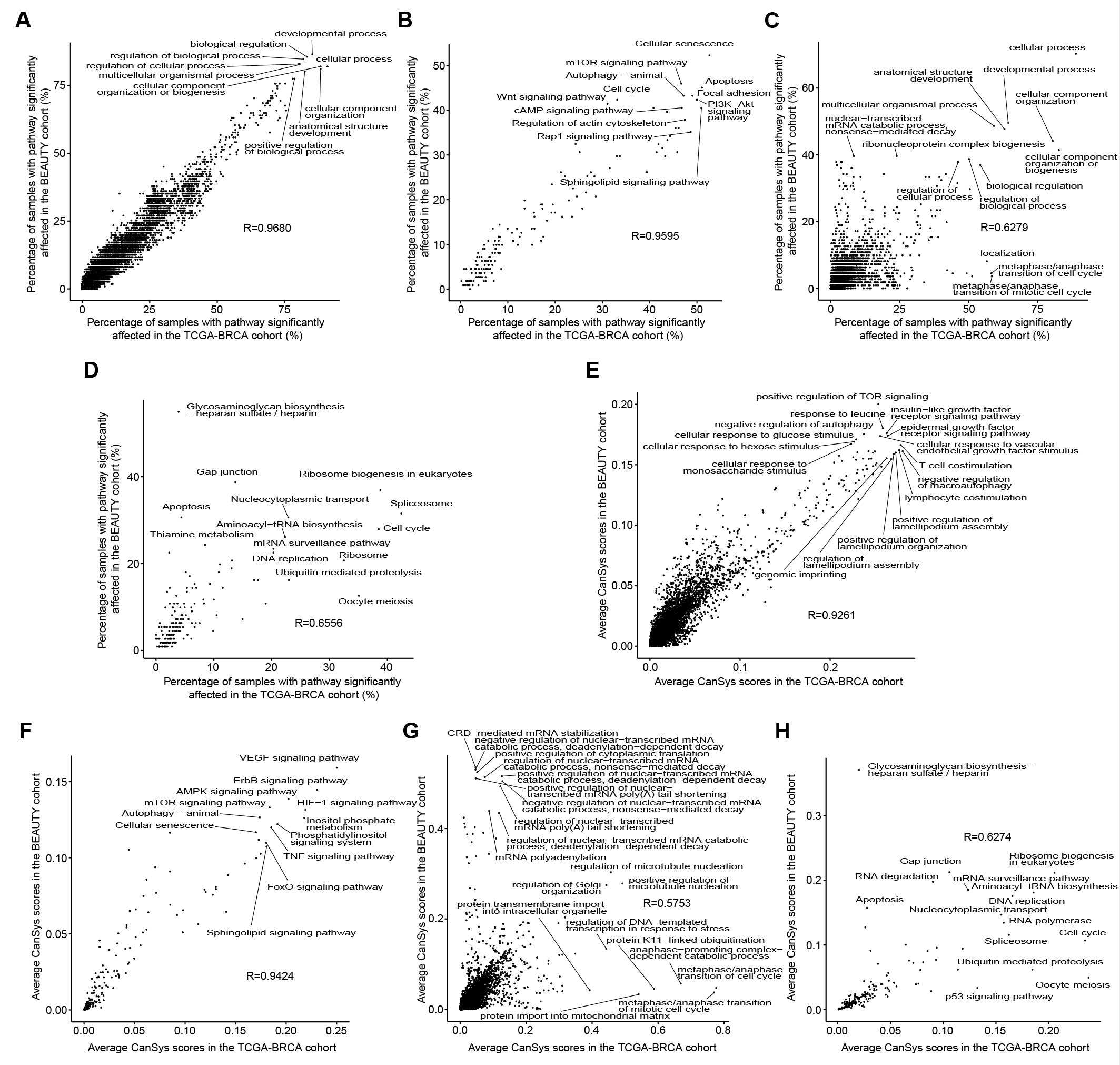


**Fig. S12: Concordance of the CanSys results between the TCGA-BRCA and BEAUTY breast cancer cohorts. A-D** Comparisons of the percentage of samples in which each pathway is significantly affected (BH adjusted *P* < 0.25) between the TCGA-BRCA and BEAUTY cohorts for somatic GO, somatic KEGG, germline GO, and germline KEGG analyses, respectively. **E-H** Comparisons of average CanSys scores between the TCGA-BRCA and BEAUTY cohorts for somatic GO, somatic KEGG, germline GO, and germline KEGG analyses, respectively. Germline variants were excluded if they had a gnomAD population allele frequency > 0.001 or a CADD score < 15. R represents the Pearson correlation coefficient computed across pathways. Annotated pathways represent the top ten most disturbed pathways in either cohort. BH: Benjamini-Hochberg procedure to adjust for multiple comparisons.


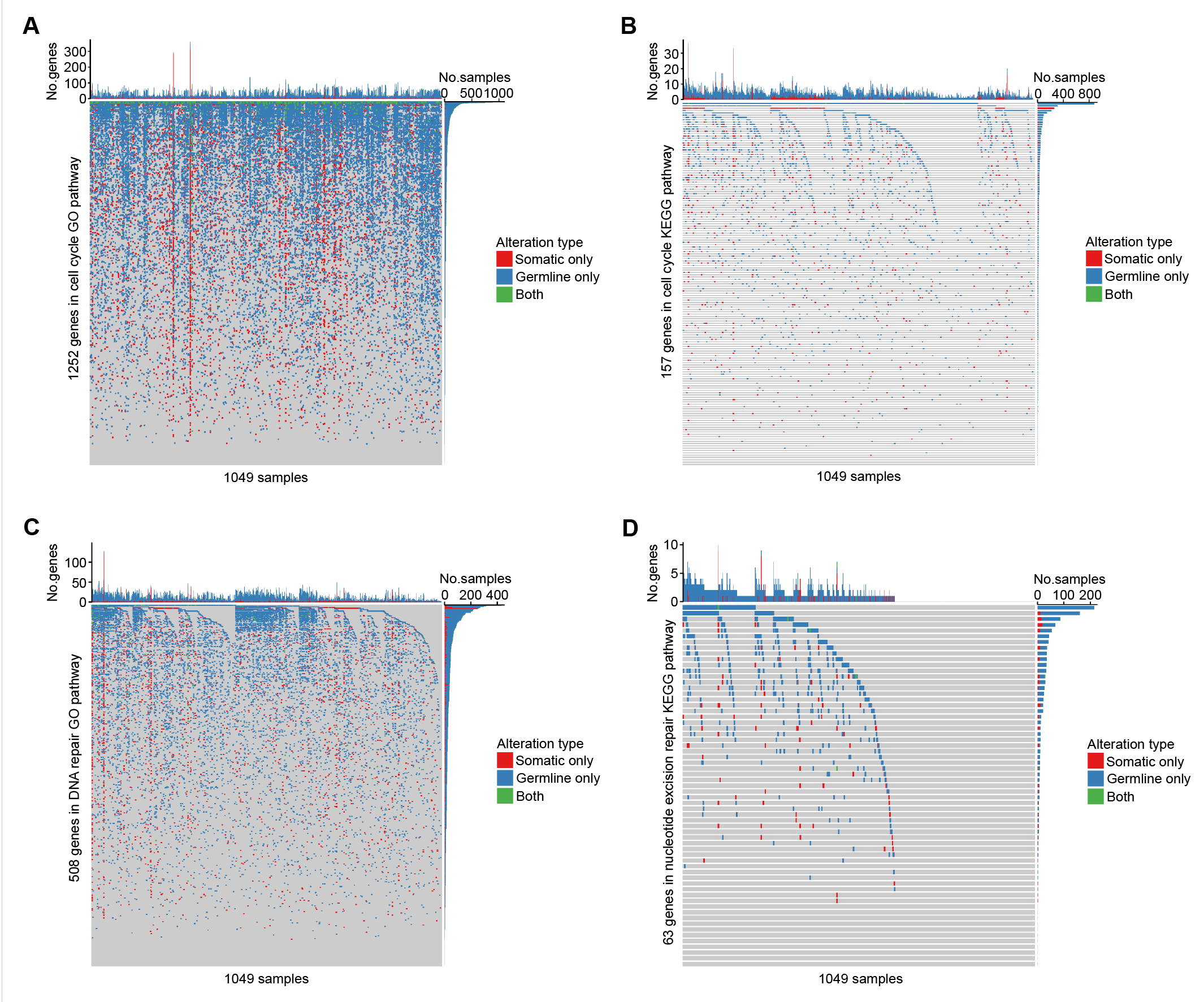


**Fig. S13: Oncoplot of gene-level alterations in 4 distinct frequently altered pathways in TCGA breast cancer samples. A** GO cell cycle pathway n=1,252. **B** KEGG cell cycle pathway n=157. **C** GO DNA repair pathway n=508. **D** KEGG nucleotide excision repair pathway n=63. Color coding indicates the source of genomic alteration (red: somatic, blue: germline, green: both). Germline variants were excluded if they had a gnomAD population allele frequency > 0.001 or a CADD score < 15.


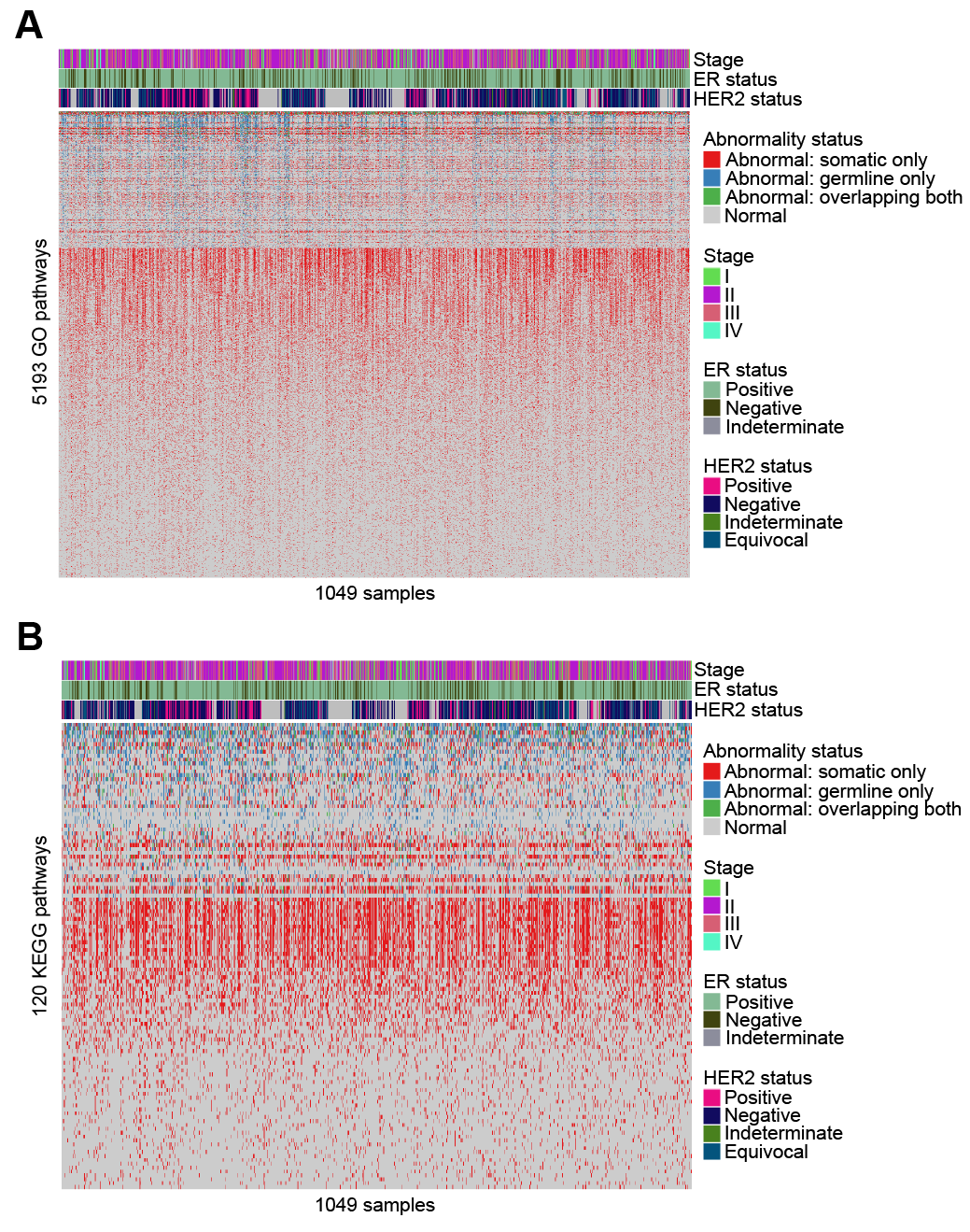


**Fig. S14: Significantly altered (BH adjusted *P* < 0.25) GO and KEGG pathways in TCGA breast cancer samples. A** Heatmap of 5,278 GO pathways affected in more than 5% of the samples. **B** Heatmap of 122 KEGG pathways affected in more than 5% of the samples. Germline variants were excluded if they had a gnomAD population allele frequency > 0.001 or a CADD score < 15. BH: Benjamini-Hochberg procedure to adjust for multiple comparisons.


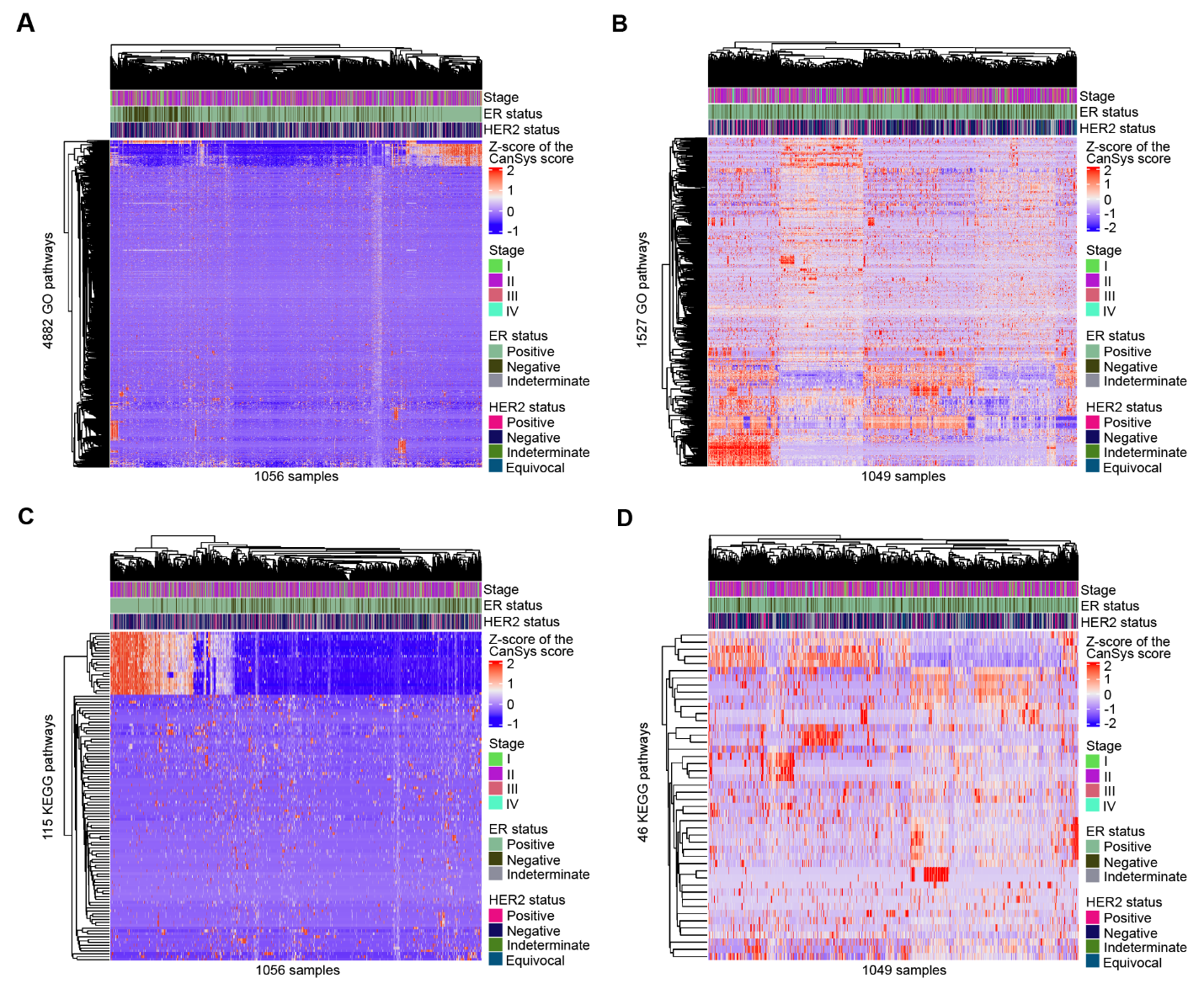


**Fig. S15: Heatmap of CanSys scores for GO and KEGG pathways across TCGA breast cancer samples. A** CanSys scores for 4,882 GO pathways affected (BH adjusted *P* < 0.25) in more than 5% of the samples at the somatic level. **B** CanSys scores for 1,068 GO pathways affected (BH adjusted *P* < 0.25) in more than 5% of the samples at the germline level. **C** CanSys scores for 115 KEGG pathways affected (BH adjusted *P* < 0.25) in more than 5% of the samples at the somatic level. **D** CanSys scores for 44 KEGG pathways affected (BH adjusted *P* < 0.25) in more than 5% of the samples at the germline level. Different numbers of GO or KEGG pathways were selected because fewer pathways are affected at the germline level. Germline variants were excluded if they had a gnomAD population allele frequency > 0.001 or a CADD score < 15. The Z-scores of the CanSys scores were capped at a range from -2 to 2. BH: Benjamini-Hochberg procedure to adjust for multiple comparisons.


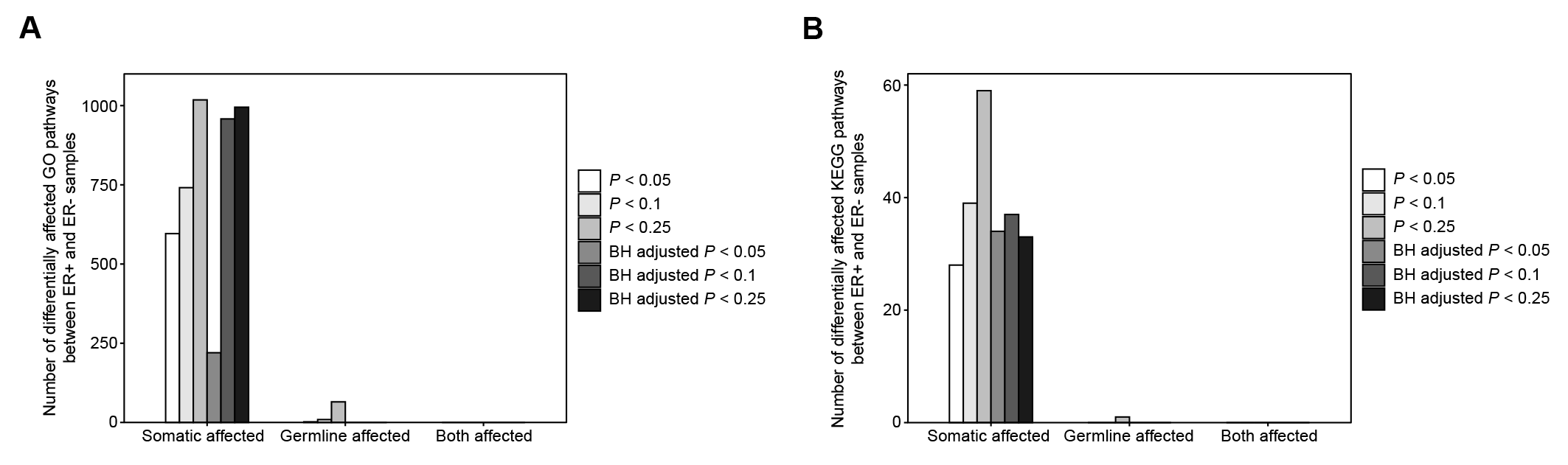


**Fig. S16**: Differentially altered biological pathways between estrogen receptor (ER)-positive and -negative breast cancers in TCGA. The number of significantly differentially affected GO (**A**) and KEGG (**B**) pathways are shown when different significance thresholds are used to define a pathway as altered at sample level. Germline variants were excluded if they had a gnomAD population allele frequency > 0.001 or a CADD score < 15. Fisher’s exact test was used to compare pathway alteration frequencies between ER groups, and BH adjusted *P* < 0.05 was considered significant. BH: Benjamini-Hochberg procedure to adjust for multiple comparisons.


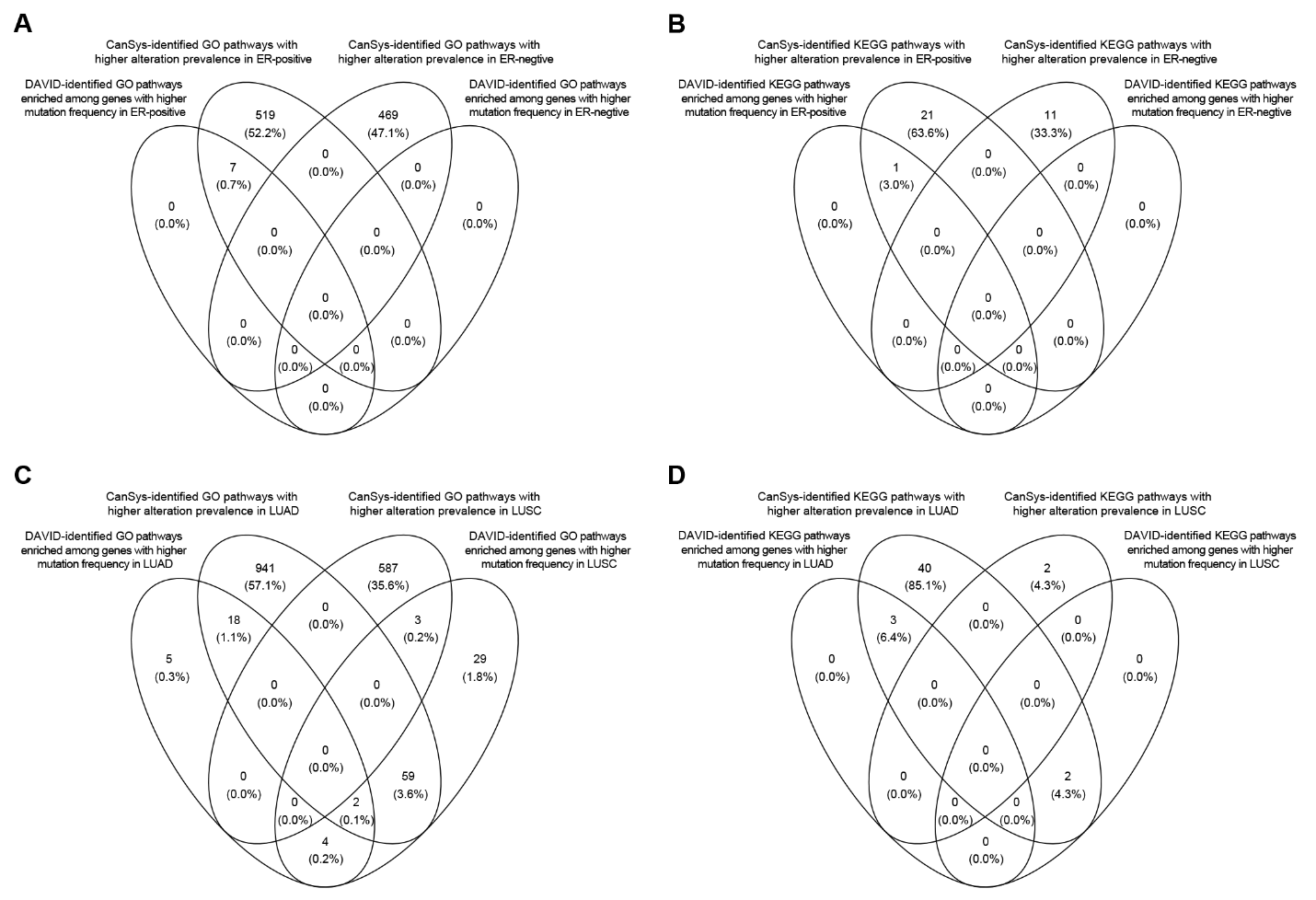


**Fig. S17: Comparison of pathway-level alterations affected by somatic variants identified by CanSys and DAVID. A** GO pathways associated with ER status in TCGA breast cancer. **B** KEGG pathways associated with ER status in TCGA breast cancer. **C** GO pathways associated with histological subtype (LUAD vs LUSC) in TCGA lung cancer. **D** KEGG pathways associated with histological subtype (LUAD vs LUSC) in TCGA lung cancer.

**
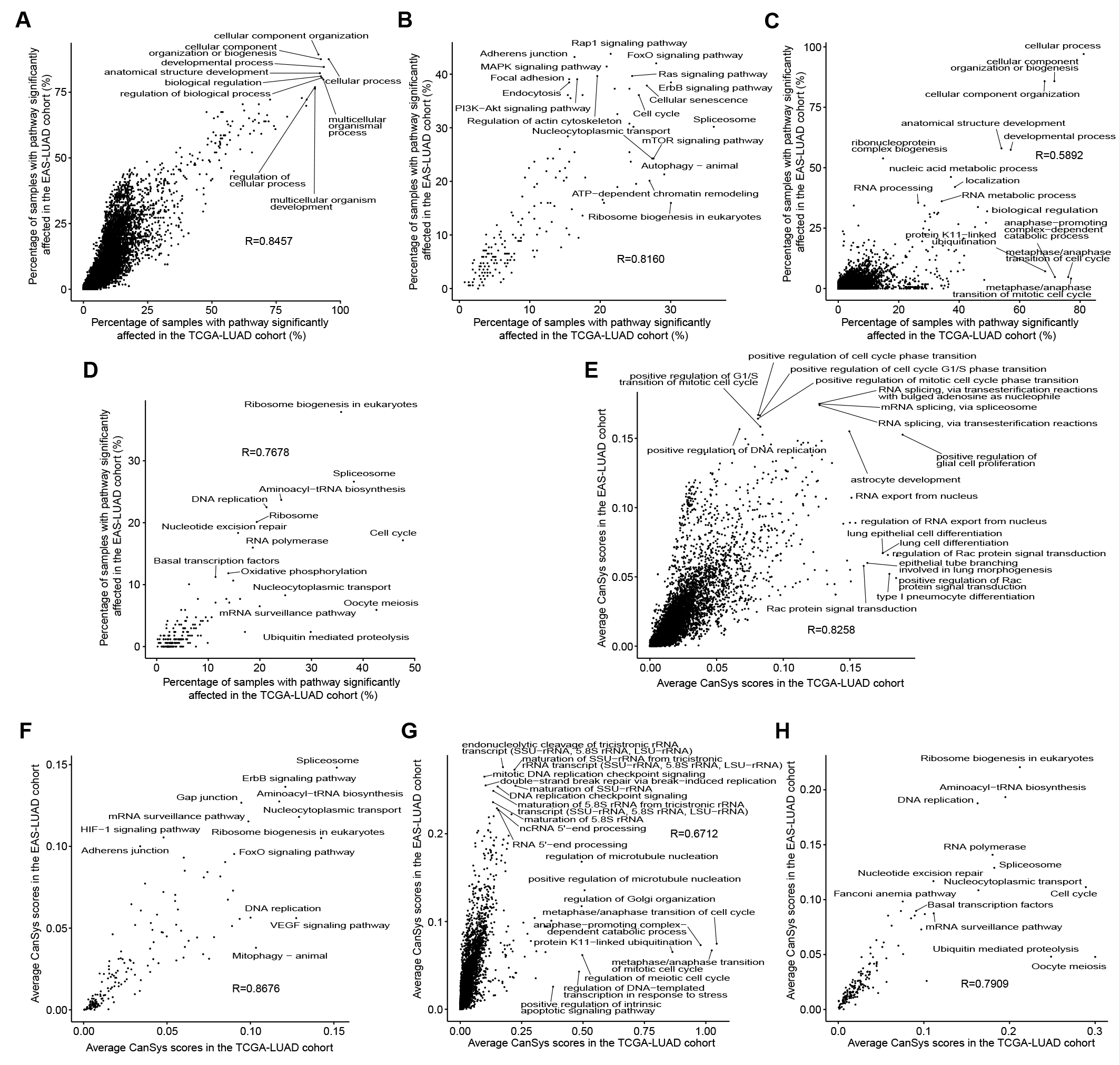
**

**Fig. S18: Concordance of the CanSys results between the TCGA-LUAD and EAS-LUAD lung adenocarcinoma cohorts. A-D** Comparisons of the percentage of samples in which each pathway is significantly affected (BH adjusted *P* < 0.25) between the TCGA-LUAD and EAS-LUAD cohorts for somatic GO, somatic KEGG, germline GO, and germline KEGG analyses, respectively. **E-H** Comparisons of average CanSys scores between the TCGA-LUAD and EAS-LUAD cohorts for somatic GO, somatic KEGG, germline GO, and germline KEGG analyses, respectively. Germline variants were excluded if they had a gnomAD population allele frequency > 0.001 or a CADD score < 15. R represents the Pearson correlation coefficient computed across pathways. Annotated pathways represent the top ten most disturbed pathways in either cohort. BH: Benjamini-Hochberg procedure to adjust for multiple comparisons.
